# Phospholipase D‐derived phosphatidic acid promotes root hair development under phosphorus deficiency by suppressing vacuolar degradation of PIN‐FORMED2

**DOI:** 10.1111/nph.16330

**Published:** 2019-12-19

**Authors:** De‐Li Lin, Hong‐Yan Yao, Li‐Hua Jia, Jin‐Fang Tan, Zhi‐Hong Xu, Wen‐Ming Zheng, Hong‐Wei Xue

**Affiliations:** ^1^ Collaborative Innovation Center of Henan Grain Crops/State Key Laboratory of Wheat and Maize Crop Science College of Life Sciences Henan Agricultural University 450002 Zhengzhou China; ^2^ National Key Laboratory of Plant Molecular Genetics CAS Center for Excellence in Molecular Plant Sciences Shanghai Institute of Plant Physiology and Ecology Chinese academy of Sciences 200032 Shanghai China; ^3^ College of Resource and Environment Henan Agricultural University 450002 Zhengzhou China; ^4^ Joint Center for Single Cell Biology School of Agriculture and Biology Shanghai Jiao Tong University 200240 Shanghai China

**Keywords:** *Arabidopsis*, phosphatidic acid (PA), SNX1, PIN2, phosphorus deficiency, vacuole

## Abstract

- Root hair development is crucial for phosphate absorption, but how phosphorus deficiency affects root hair initiation and elongation remains unclear.
- We demonstrated the roles of auxin efflux carrier PIN‐FORMED2 (PIN2) and phospholipase D (PLD)‐derived phosphatidic acid (PA), a key signaling molecule, in promoting root hair development in *Arabidopsis thaliana* under a low phosphate (LP) condition.
- Root hair elongation under LP conditions was greatly suppressed in *pin2* mutant or under treatment with a PLDζ2‐specific inhibitor, revealing that PIN2 and polar auxin transport and PLDζ2‐PA are crucial in LP responses. PIN2 was accumulated and degraded in the vacuole under a normal phosphate (NP) condition, whereas its vacuolar accumulation was suppressed under the LP or NP plus PA conditions. Vacuolar accumulation of PIN2 was increased in *pldζ2* mutants under LP conditions. Increased or decreased PIN2 vacuolar accumulation is not observed in *sorting nexin1* (*snx1*) mutant, indicating that vacuolar accumulation of PIN2 is mediated by SNX1 and the relevant trafficking process. PA binds to SNX1 and promotes its accumulation at the plasma membrane, especially under LP conditions, and hence promotes root hair development by suppressing the vacuolar degradation of PIN2.
- We uncovered a link between PLD‐derived PA and SNX1‐dependent vacuolar degradation of PIN2 in regulating root hair development under phosphorus deficiency.

## Introduction

P_i_ is an essential macronutrient that plays a crucial role in multiple physiological processes and metabolic pathways in plants. P_i_ availability is a major factor limiting crop production. As the P_i_ in soils has low availability and limited mobility, plants have evolved complex mechanisms in response to phosphorus (P) deficiency, including morphological and physiological responses (Raghothama, 1999; Péret *et al.*, 2011; Plaxton & Tran, 2011). In *Arabidopsis thaliana*, P_i_ deficiency inhibits root apical meristem activity through reduced cell division and the loss of a quiescent center, while inducing lateral root formation and growth (Sánchez‐Calderón *et al.*, 2005). P_i_‐deficiency‐promoted root hair number and length enhance overall root absorption capacity and efficiency (Bates & Lynch, 2000).

A major metabolic event in the plant response to P_i_ deficiency is membrane lipid remodeling, in which lipid metabolic and signaling pathways are activated to increase the levels of non‐P‐containing galactolipids and decrease the levels of phospholipids to scavenge and reserve internal P_i_ for other cellular needs (Andersson *et al.*, 2003; Nakamura *et al.*, 2009; Shimojima & Ohta, 2011; Nakamura, 2013; Pant *et al.*, 2015). In *Arabidopsis*, the genes encoding phospholipase Dζ2 (*PLDζ2*) and nonspecific phospholipase C4 (*NPC4*) are highly induced in response to P_i_ deficiency (Nakamura *et al.*, 2005; Cruz‐Ramirez *et al.*, 2006; Li *et al.*, 2006). PLDζ2 hydrolyzes phosphatidylcholine and phosphatidylethanolamine to produce diacylglycerol, which is later converted to digalactosyldiacylglycerol, while the free P_i_ is recycled and utilized for other processes (Cruz‐Ramírez *et al.*, 2006). PLDζ2 and NPC4 play distinct roles in root hair growth and development in response to P_i_ deprivation: PLDζ2 negatively modulates root hair density and length, whereas NPC4 promotes root hair elongation (Su *et al.*, 2018). PLDζ2 and its product, phosphatidic acid (PA), also function in root growth and auxin responses by regulating the cycling of the auxin efflux carrier PIN‐FORMED2 (PIN2; Li & Xue, 2007). Phospholipase D (PLD)‐derived PA functions in root hair patterning and growth by binding to distinct target proteins and regulating their activity and subcellular localization (reviewed by Yao & Xue, 2018).

The phytohormone auxin plays crucial roles in plant responses to P_i_ deficiency by regulating primary root, lateral root, and root hair development (López‐Bucio *et al.*, 2002; Nacry *et al.*, 2005; Kapulnik *et al.*, 2011; Martín‐Rejano *et al.*, 2011). The increased auxin sensitivity of plants under a low phosphate (LP) condition appears to be directly associated with enhanced lateral root initiation and emergence (López‐Bucio *et al.*, 2002; Pérez‐Torres *et al.*, 2008). The auxin level of root tips is upregulated – in particular, that of root hair zones is significantly increased – under LP conditions (Nacry *et al.*, 2005; Bhosale *et al.*, 2018), which is consistent with that the auxin biosynthesis gene (*TRYPTOPHAN AMINOTRANSFERASE OF ARABIDOPSIS1*, *TAA1*) is significantly upregulated under LP conditions. Under LP conditions, TAA1 (involved in auxin biosynthesis) promotes root hair development by mediating auxin biosynthesis (the *taa1* mutant shows shorter root hairs under LP conditions), whereas *DIOXYGENASE FOR AUXIN OXIDATION1* (*DAO1*), the auxin inducible IAA‐degrading enzyme) inhibits root hair development by increasing auxin degradation (the *dao1.2D* mutant with increased *DAO1* expression shows shorter root hairs under LP conditions) (Bhosale *et al.*, 2018; Giri *et al.*, 2018), indicating that altered biosynthesis/metabolism of auxin is essential for root hair growth and development in response to P_i_ deficiency.

Auxin transport from the root apex to the differentiation zone through influx and efflux carriers (such as AUX1 and PIN) promotes auxin‐dependent root hair growth in response to P_i_ deficiency. Auxin is mobilized via AUX1 to the root hair differentiation zone to promote root hair elongation, and *aux1* mutant presents shorter root hairs under LP conditions (Bhosale *et al.*, 2018). The subcellular localization and accumulation of PIN proteins are important for the formation of the auxin gradient that is crucial to plant development (Petrasek & Friml, 2009). PIN2 localizes at the cortex and epidermal cells of root tips and mediates the transport of auxin from the root tip toward the elongation zone, creating the auxin gradient in roots together with other transporting proteins (Friml *et al.*, 2002; Petrášek *et al.*, 2006; Wiśniewska *et al.*, 2006). Whether PIN2 functions in the root hair development under P_i_ deficiency is still unclear. The activity and proper localization of PIN2 are regulated by a serine/threonine (Ser/Thr) protein kinase PINOID (PID) and a protein phosphatase 2A (PP2A; Michniewicz *et al.*, 2007). PIN2 stability is regulated by various factors, and PIN2 accumulates in the vacuole under light–dark transition (Kleine‐Vehn *et al.*, 2008; Laxmi *et al.*, 2008). Further studies showed that PIN2 accumulation in the vacuole is regulated by inositol 1,4,5‐trisphosphate (IP_3_) and high‐temperature treatment, a process mediated by SORTING NEXIN (SNX)‐containing endosomes (Hanzawa *et al.*, 2013; Chu *et al.*, 2016).

SNXs are endosomal regulatory proteins first discovered in human cells, where they affect the sorting of the epidermal growth factor receptor for degradation in lysosomes (Kurten *et al.*, 1996). Mammals have *c.* 30 SNX family members, whereas *Arabidopsis* has only six: SNX1, SNX2a, SNX2b, and three unexplored proteins (SNX3, SNX4 and SNX5). SNX1 recruits SNX2a and SNX2b to the endosome by forming SNX1–SNX2 dimers (Pourcher *et al.*, 2010; Jaillais *et al.*, 2016; Heucken & Ivanov 2018). SNX proteins function in various physiological processes, especially responses to environmental stimuli (Hanzawa *et al.*, 2013; Ivanov *et al.*, 2014; Li *et al.*, 2018; Salanenka *et al.*, 2018). *snx1* seedlings exhibit pronounced growth arrest, including shortened primary roots on low‐sucrose medium (Kleine‐Vehn *et al.*, 2008; Hirano *et al.*, 2015). Salt stress induces the expression of *SNX1*, thereby regulating nitric oxide (NO) synthase activity and NO accumulation. The NO synthase‐like activity and the expression of some superoxide dismutases are inhibited in *snx1* mutant (Li *et al.*, 2018). At high temperature, SNX1 regulates intracellular auxin levels by mediating the transport of PIN2 from late endosomes to the plasma membrane (Hanzawa *et al.*, 2013).

In yeast, the SNX1–retromer complex is required to transport proteins from the prevacuolar compartment (PVC) to the trans‐Golgi network (Seaman, 2005). *Arabidopsis* SNX1 is located in the PVC and is involved in the vacuolar sorting of PIN2 at the PVC. In *snx1* seedlings, PIN2 shows reduced abundance at the plasma membrane and accumulates in the vacuoles (Kleine‐Vehn *et al.*, 2008). The endocytosis of SNX1 is promoted by IP_3_, leading to reduced SNX1 accumulation at the plasma membrane, thereby suppressing the degradation of membrane proteins (Chu *et al.*, 2016), providing a mechanism for the regulation of the vacuolar degradation of membrane proteins. In addition, SNX1 mediates the homeostasis of PIN1 and PIN2 by directly interacting with biogenesis of lysosome‐related organelles complex 1 (Cui *et al.*, 2010).

Although crosstalk between low P and auxin has been reported, little is known about the underlying mechanism. Here, through systematic genetic and biochemical analyses, we demonstrate that PLDζ2‐derived PA directly binds to SNX1 to promote its accumulation at the plasma membrane, resulting in the suppressed endocytosis and vacuolar degradation of PIN2, thereby stimulating the root hair and plant growth under LP conditions.

## Materials and Methods

### Plant materials and growth conditions

Seedlings Col‐0, pPIN2:PIN2‐green fluorescent protein (GFP), p35S:SNX1‐mcherry, pSNX1:SNX1‐red fluorescent protein (RFP), pSNX2a:SNX2a‐GFP, DR5:GFP, DR5:β‐glucuronidase (GUS), *pin2* (*eir1‐1*), *pldζ2* (Salk_094369), *snx1* (SALK_033351), pPIN2:PIN2‐GFP in *pldζ2* (Salk_094369), pPIN2:PIN2‐GFP in PLDζ2‐ox, and pPIN2:PIN2‐GFP in *snx1* (SALK_033351) background were used. The sterilized seeds of *Arabidopsis thaliana* were stratified at 4°C for 2 d, then evenly spread in ½ Murashige & Skoog (½MS) with 2% sucrose and germinated in phytotron with a 16 h : 8 h, light : dark, cycle (23°C). Seven‐day‐old seedlings were transferred to soil and grown in phytotron. The ½MS normal condition medium contains 0.305 g l^−1^ MS basal salt mixture without nitrogen (N), P, and potassium (K) (PhytoTechnology Laboratories, Shawnee Mission, KS, USA), 18.79 mM potassium nitrate, 20.61 mM ammonium nitrate, 1.25 mM potassium dihydrogen phosphate (KH_2_PO_4_), 0.43 g l^−1^ Mes, 20 g l^−1^ sucrose, and 8.5 g agar, pH 5.85. The ½MS low‐P medium contains 0.625 mM potassium sulfate instead of 1.25 mM KH_2_PO_4_.

Full‐length complementary DNA (cDNA) of SNX1 was amplified using primers (forward: 5′‐CGGGATCCATGGAGAGCACGGAGCAGCCGA‐3′; reverse: 5′‐GCGTCGACGACAGAATAAGAAGCTTCAAGT‐3′) and then subcloned into pCambia1300‐mCherry vector. Confirmed construct (p35S:SNX1‐mCherry) was transformed into *Arabidopsis* by the floral dip method.

### Expression of recombinant protein, protein extraction, and immunoblotting analysis

Full‐length cDNA of SNX1 was amplified using primers (forward: 5′‐GCGGATCCGATGGAGAGCACGGAGCAGCCGA‐3′; reverse: 5′‐CCGAGCTCCGACAGAATAAGAAGCTTCAAGT‐3′) and subcloned into pET51b (Novagen, Madison, WI, USA). Confirmed construct was transformed into *Escherichia coli* Rosetta (DE3) and SNX1 protein expression was induced by supplementing with isopropyl‐β‐d‐1‐thiogalactopyranoside (0.1 mM) for 10–16 h at 16°C. Histidine (His)‐tagged SNX1 was purified using nickel nitrilotriacetic acid agarose gel electrophoresis according to the manufacturer's protocol (Novagen).

Plant tissues were collected and homogenated in protein extraction buffer (20 mM Tris hydrochloride (Tris‐HCl), pH7.5, 150 mM sodium chloride (NaCl), 0.5% Tween‐20, 1 mM EDTA, 1 mM dithiothreitol (DTT)) containing a protease inhibitor cocktail (Roche, Basel, Switzerland) on ice for 30 min, centrifuged at 12 000 ***g*** for 15 min, and the supernatant was collected as the total protein. For the immunoblot of PIN2‐GFP, root tips of treated seedlings were harvested to extract the total proteins, and analyzed using anti‐GFP antibody (Abcam, Cambridge, UK), which were quantified by actin (Abcam).

To extract the membrane proteins, the seedlings were ground in liquid nitrogen, and added in grinding buffer (20 mM Tris‐HCl, pH 8.8, 150 mM NaCl, 1 mM EDTA, 20% glycerol, protease inhibitors) for 30 min on ice, centrifuged (6000 ***g***) for 30 min, and then centrifuged (100 000 ***g***) for 60 min. The pellets were resuspended in dissolving buffer (10 mM Tris‐HCl, pH 7.5, 150 mM NaCl, 1 mM EDTA, 10% glycerol, 1% Triton X‐100, protease inhibitors); for details, see Gao *et al.* (2013) and Chu *et al.* (2016). Extracted membrane proteins were quantified by bicinchoninic acid and Coomassie Brilliant Blue staining. Equal amounts of protein were separated by 10% sodium dodecyl sulfate polyacrylamide gel electrophoresis and detected by immunoblotting.

Immunoblotting analysis was performed according to previous description (Tan *et al.*, 2013). The activities of immunoglobulin G alkaline phosphatase (AP)‐conjugated secondary antibody (Santa Cruz Biotechnology, Delaware Ave Santa Cruz, CA, USA) or horseradish peroxidase‐conjugated secondary antibody (Abcam) were detected using a BCIP/NBT kit (Invitrogen, Waltham, MA, USA) or by ECL Western blotting substrate (Tanon, Pujiang High‐tech Park, Shanghai, China), respectively. The brand density was measured and calculated by ImageJ (NIH, Bethesda, MD, USA).

### Lipid–protein blotting and liposomal binding

PA–protein binding was assayed by using lipid–protein blotting. PA was dissolved in chloroform and different concentrations of PA were spotted onto a nitrocellulose membrane and dried at room temperature for *c.* 2 h in darkness. The nitrocellulose membranes contained different lipids (PIP Strips™ membranes; P23751) were purchased from ThermoFisher (Waltham, MA, USA). The nitrocellulose membranes were incubated for 1 h at room temperature in 3% fatty‐acid‐free BSA (w/v) in the TBST (Tris‐buffered saline, Tween 20) solution. After washing with TBST three times, the membranes were incubated at 4°C overnight with TBST containing the purified protein of SNX1. Then, after washing three times with TBST, the membranes were then incubated at room temperature for 4 h with anti‐His tag (Abcam) in TBST, followed by three washes with TBST and then incubated at room temperature for 1 h with secondary antibody. The AP activity was detected using a BCIP/NBT kit (Invitrogen).

Liposomal binding was performed according to a previously described method (Yao *et al.*, 2013) with slight modifications. Dioleoyl PC and dioleoyl PA (both obtained from Avanti Polar Lipids, Alabaster, Alabama, AL, USA) were dissolved in chloroform in a 3 : 1 molar ratio and then dried under N. Dried lipids were rehydrated in extrusion buffer (250 mM raffinose, 25 mM Tris‐HCl pH 7.5, 1 mM DTT) at 42°C for 1 h. The extrusion buffer containing lipids was extruded repeatedly by the liposome extruder through the polycarbonate membrane (0.2 μm pore size) to produce an optically clear suspension of small unilamellar liposomes according to the manufacturer's instructions (Avanti Polar Lipids). The small unilamellar liposomes were diluted with three volumes of binding buffer (25 mM Tris‐HCl pH 7.5, 1.25 mM potassium chloride, 0.5 mM EDTA, 1 mM DTT). The pellets were harvested by centrifugation (100 000 ***g***) for 40 min and then resuspended in binding buffer to generate the stock (3.2 mM). Different concentrations of liposomes were incubated with the purified proteins at 25°C for 45 min and the liposomes‐containing proteins were centrifuged (14 000 ***g***) for 30 min. The pellets were washed twice with binding buffer and the supernatant transferred to a new tube. The protein remaining in the supernatant was precipitated by the addition of 1 : 10 (v/v) of 100% trichloroacetic acid in ice for 30 min and then centrifuged (14 000 ***g***) for 10 min. The pellets were then washed twice with acetone and the liposome‐bound proteins in the supernatant detected by immunoblotting analysis.

### Chemical treatments and histochemical analysis of β‐glucuronidase activities

Brefeldin A (BFA; Yeasen Biotechnology, Pudong New Area, Shanghai, China), dissolved in dimethyl sulfoxide as 40 mM stock, was diluted to 40 µM for treatment. Wortmannin (Yeasen Biotechnology), dissolved in dimethyl sulfoxide as 34 mM stock, was diluted to 16.5 µM for treatment. PA (50 mM, Sigma‐Aldrich, MO, USA) was dissolved in 100% ethanol and the concentration used was 50 µM. PLD inhibitor 1‐butanol (0.2%, w/w) was applied with 2‐butanol as control (both obtained from Sigma‐Aldrich). The working concentration of PLD2 inhibitor (VU0285655‐1; Avanti Polar Lipids) was 0.2 μM. FM 4‐64 (ThermoFisher), dissolved in sterile water, was diluted to 2 µM for treatment.

For LP treatment, seedlings were grown on ½MS for 4 d and then transferred to medium containing normal P_i_ (1.25 mM, normal phosphate (NP)) or low P_i_ (0 mM, LP) for 3, 5, and 7 d for observation. For treatment with PA, 1‐butanol, or 2‐butanol, the seedlings were grown on ½MS for 6 d and then transferred into NP or LP liquid medium containing PA (50 µM), 1‐butanol, or 2‐butanol (0.2%). Seedlings were harvested to extract the membrane proteins, and the degradation of PIN2 in the vacuole was observed at different times.

For PLD2 inhibitor or wortmannin treatment, the seedlings were germinated on ½MS for 4 d and then transferred to NP or LP medium containing PLD2 inhibitor (200 nM), wortmannin (16.5 μM), or dimethyl sulfoxide (DMSO) for different times.

To observe the endocytosis of PIN2‐GFP, SNX1‐RFP and SNX2a‐GFP, the seedlings were grown on ½MS for 6 d and then transferred to LP or NP liquid medium containing BFA. BFA inhibits the vesicle transport from endosomes to the plasma membrane and leads to the endosomes aggregating (so called BFA bodies); the number of BFA bodies per cell was observed and counted after treatment for different times by using a confocal microscope. For exocytosis analysis, seedlings were transferred in liquid ½MS containing BFA for 1.5 h and then washed in LP or NP liquid medium to count the number of BFA bodies per cell at different times.

For histochemical analysis of GUS reporter enzyme activity, the DR5‐GUS seedlings were grown under the NP condition, the LP condition with PLD2 inhibitor (200 nM), wortmannin (16.5 μM), or DMSO for 5 d, then incubated in the GUS solution for 12 h at 37°C, and then observed according to a previous description (Tang *et al.*, 2016).

### Morphological analysis

The length of primary root was measured using the ImageJ program. Photographs of root hairs were taken using the differential interference contrast microscope, and the length and number of root hairs were measured using the ImageJ program.

### Confocal laser microscopy observation

An LSM‐880 laser‐scanning confocal microscope (Zeiss, Jena, Germany) and Fluoview‐FV10i laser‐scanning confocal microscope (Olympus, Tokyo, Japan) were used. GFP or monomeric RFP fluorescence was excited by the 488 nm argon laser or the 561 nm He–Ne laser, respectively. Settings of the confocal microscope were the same for all image capture processes.

## Results

### PIN2 deficiency results in defective root hair elongation under phosphorus deficiency

Root system architecture is strongly affected by the soil environment, especially nutrient levels. Under LP conditions, primary root growth is significantly suppressed, whereas the initiation and elongation of lateral roots and root hairs are promoted. The biosynthesis and transport of the phytohormone auxin are critical in promoting root hair growth in response to LP. Since the auxin efflux carrier PIN2 plays crucial roles in root hair development, we investigated whether polar auxin transport is involved in altered root hair growth under LP conditions, as well as the underlying regulatory mechanism.

**Figure 1:**
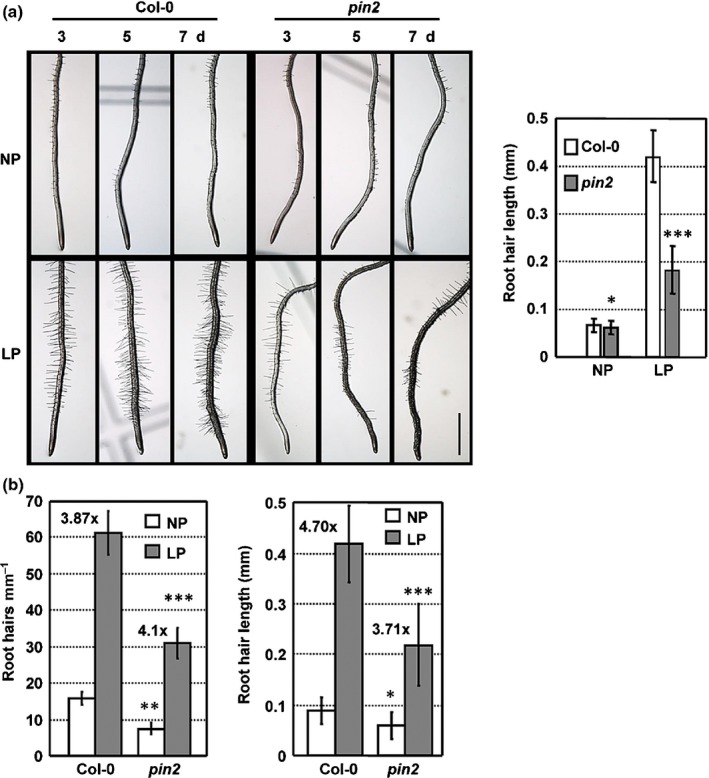
Loss‐of‐function *pin‐formed2* (*pin2*) plants exhibit fewer, shorter root hairs under low phosphate (LP) conditions. (a) *Arabidopsis thaliana* Col‐0 and *pin2* seedlings were grown in ½ Murashige & Skoog for 4 d and transferred to normal phosphate (NP; 1.25 mM P_i_) or LP conditions (0 mM P_i_) for 3, 5 and 7 d. Root hair number and length were observed (left) and root hair length of seedlings after treatment for 5 d was measured (right). Bar, 1 cm. The experiments were performed with three biological repeats, and data are presented as means ± SE (*n *> 50). Statistical analysis was performed by two‐tailed Student's *t*‐test (*, *P* < 0.05; ***, *P* < 0.001, compared with Col‐0 under same condition). (b) Root hair number and length of Col‐0 and *pin2* seedlings under NP or LP conditions for 7 d. Numbers above the bars indicate the increased fold under LP vs NP conditions. The experiments were performed with three biological repeats, and data are presented as means ± SE (*n *> 50). Statistical analysis was performed by two‐tailed Student's *t*‐test (*, *P* < 0.05; **, *P* < 0.01; ***, *P* < 0.001, compared with Col‐0 under same condition).

By using a specific nutrient system (see the Materials and Methods section), studies revealed that root hair length and numbers are significantly increased in the wild‐type (WT) under LP conditions (Fig. 1a,b), whereas these responses were almost completely disrupted in the *Arabidopsis pin2* mutant; in particular, the root hair length was greatly reduced (Fig. 1a). Fewer root hairs were present in *pin2* than in WT seedlings under both NP and LP conditions (Fig. 1b). Although the inhibition of primary root growth and increased lateral root number were not significantly altered (Supporting Information Fig. S1), root hair elongation was suppressed in *pin2* seedlings under LP (Fig. 1b), indicating that PIN2‐mediated polar auxin transport is involved in root hair development under P deficiency.

### PIN2 degradation in vacuoles is suppressed under phosphorus deficiency or treatment with phospholipase D‐derived phosphatidic acid

**Figure 2:**
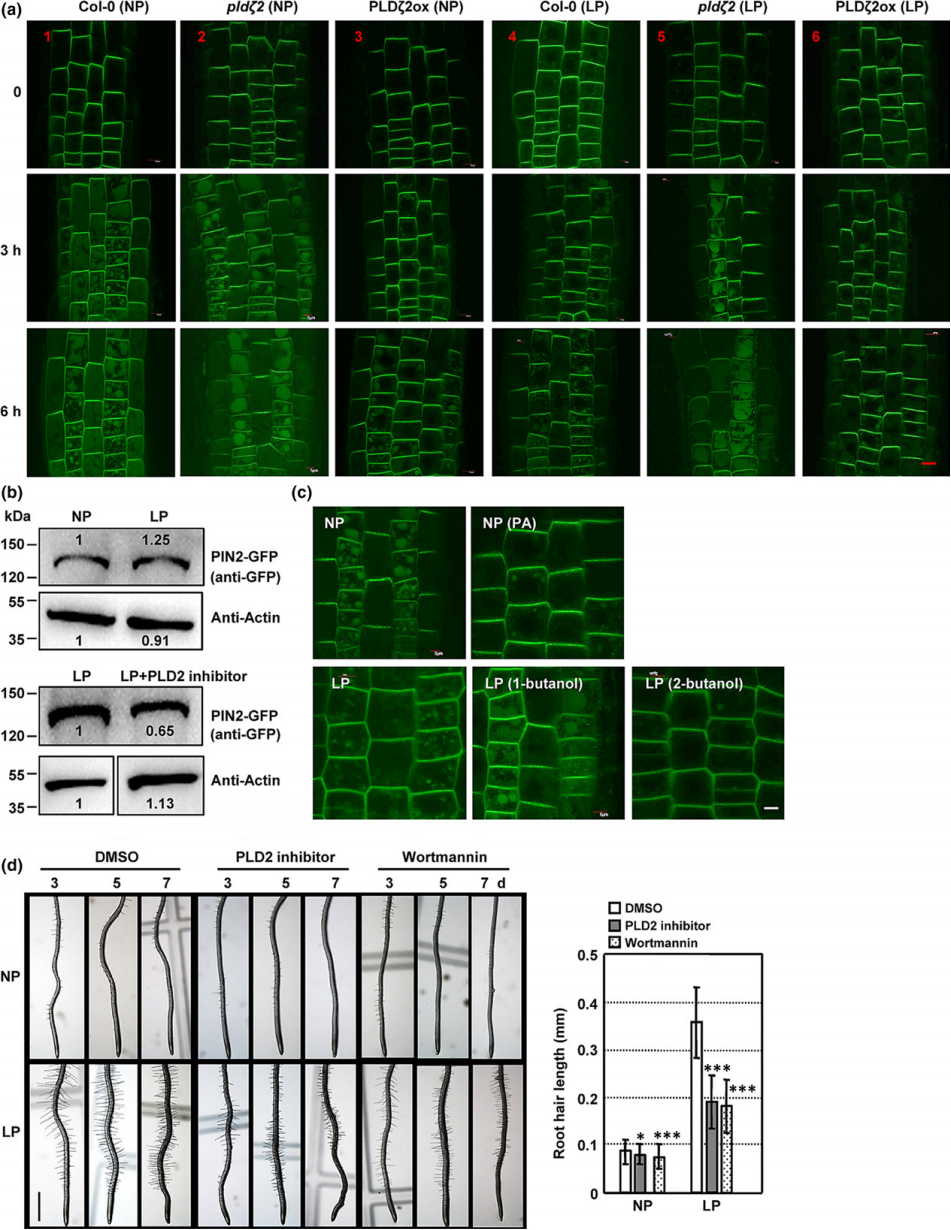
PIN‐FORMED2 (PIN2) protein degradation in vacuoles is suppressed by low phosphate (LP) conditions or phospholipase Dζ2 (PLDζ2)‐derivated phosphatidic acid (PA). (a) PIN2 accumulates and is degraded in vacuoles under normal phosphate (NP) conditions (1.25 mM P_i_) (1), but this process is suppressed under LP conditions (0 mM P_i_) (4) or PLDζ2 overexpression (3). Compared with *Arabidopsis thaliana* Col‐0, PIN2 protein degradation in vacuoles is enhanced in *pldζ2* plants under both NP (2) and LP conditions (5) and suppressed in *PLDζ2*ox plants under LP conditions (6). Fluorescence of the PIN2‐green fluorescent protein (GFP) fusion protein in roots of Col‐0, *pldζ2* or PLDζ2ox plants under NP or LP conditions was observed and representative images are shown. Bar, 5 μm. (b) Immunoblot analysis of PIN2‐GFP protein abundance at root tips under NP or LP conditions (top), or LP conditions under treatment with PLD2 inhibitor (bottom). PIN2‐GFP seedlings were grown on ½ Murashige & Skoog for 3 d and transferred to NP or LP conditions, or LP with PLD2 inhibitor (200 nM) for 7 d. Total proteins from root tips of treated seedlings were extracted and analyzed by using the GFP antibody again. Actin was used as a loading control. Band density was measured using ImageJ. (c) PA treatment (50 μM) decreases PIN2 accumulation and degradation in the vacuole under NP conditions, whereas 1‐butanol treatment (0.2%, 2‐butanol was used as a control) promotes PIN2 accumulation and degradation in the vacuole under LP conditions. Fluorescence of the PIN2‐GFP fusion protein was observed, and representative images are shown. Bar, 5 μm. (d) Root hair number and length of Col‐0 seedlings grown under NP or LP conditions in the presence of PLD2 inhibitor (0.2 μM) or wortmannin (16.3 μM, dimethyl sulfoxide (DMSO) was used as a control) for 3, 5 and 7 d were observed (left) and root hair length of seedlings under treatment for 5 d were measured. Bar, 1 cm. The experiments were biologically repeated three times and data are presented as means ± SE (*n* > 50) (right). Statistical analysis was performed by two‐tailed Student's *t*‐test (*, *P* < 0.05; ***, *P* < 0.001, compared with control treatment).

Membrane‐localized PIN2 is degraded in the vacuole. Given that, under LP conditions, root hair elongation is reduced and auxin transport from the root tip to the shoot is blocked in *pin2* seedlings but *PIN2* expression is not significantly altered (Kumar *et al.*, 2015; Bhosale *et al.*, 2018), we examined the PIN2 degradation under both the NP and LP conditions. Indeed, observation of the fluorescence of PIN2‐GFP fusion protein revealed the increased accumulation of PIN2 in the vacuole under NP conditions (Fig. 2a), visualized by using the endocytic dye FM4‐64, which labels the tonoplast (Fig. S2; Kleine‐Vehn *et al.*, 2008), which was consistent with previous findings (Kleine‐Vehn *et al.*, 2008; Laxmi *et al.*, 2008). By contrast, PIN2 accumulation in the vacuole was strongly suppressed under LP conditions (Fig. 2a). Further immunoblotting analysis using total proteins extracted from root tips confirmed the increased level of PIN2‐GFP under LP conditions (Figs 2b (top), S3), suggesting that P deficiency inhibits PIN2 degradation in the vacuole. By using marker lines DR5::GFP and DR5::GUS, observations revealed the increased signals in root tips and elongation zones where root hair initiates (Fig. S4), indicating the increased auxin level in roots under LP conditions, which was consistent with the recent reports (Bhosale *et al.*, 2018).

PLDζ2 produces PA, a key signaling molecule involved in various physiological processes, by directly binding to various target proteins (Yao & Xue, 2018). PLDζ2 and PA mediate auxin transport and distribution through regulating the cycling of PIN2 (Li & Xue, 2007). Under salt stress, PLD‐derived PA binds with PID and increases the PID‐dependent phosphorylation of PIN2 to activate auxin efflux and alter auxin accumulation, resulting in the promoted root growth (Wang *et al.*, 2019). PA also binds with the scaffolding A1 subunit of protein phosphatase 2A (PP2AA1) to regulate the PP2A‐mediated PIN1 dephosphorylation and hence the auxin distribution (Gao *et al.*, 2013). As *PLDζ2* transcription is strongly induced in both roots and shoots under P_i_ starvation, and this induction is fairly specific relative to deficiencies of other nutrients, including iron, K, sulfur, and N (Cruz‐Ramírez *et al.*, 2006), we investigated whether PLDζ2 is involved in mediating the vacuolar degradation of PIN2 under LP conditions. Analysis by observing the PIN2‐GFP fluorescence showed that PIN2 accumulation in vacuoles was increased in *pldζ2* mutant, even under LP conditions (Fig. 2a), but that was strongly suppressed in PLDζ2‐overexpressing lines under NP or LP conditions (Fig. 2a). These results suggested that PLDζ2 played a crucial role in regulating the vacuolar degradation of PIN2 under P_i_ starvation.

We then treated plants with the PLD inhibitor 1‐butanol – which reduces cellular PA levels by promoting the production of phosphatidylalcohols instead of PA from PLD (Munnik *et al.*, 1995) – to evaluate the effects of PA on the degradation of PIN2 under LP conditions. The vacuolar accumulation of PIN2 was clearly increased in treatment with 1‐butanol under LP conditions (Fig. 2c), which was consistent with the findings for *pldζ2* seedlings under LP conditions (Fig. 2a). Conversely, under NP conditions, treatment with PA suppressed the vacuolar accumulation of PIN2 (Fig. 2c). These results indicate that PLDζ2‐derived PA mediated the response to the LP condition and regulated the accumulation of PIN2 in the vacuole. However, further observation of the root development of *pldζ2* seedlings under P deficiency showed that the primary root length, lateral root numbers, and number and length of root hairs of *pldζ2* and PLDζ2‐overexpressing lines were similar to that of WT under NP or LP conditions (Fig. S5). This is possibly due to the functional redundancy of PLD family members. Indeed, transcription levels of some members of the PLD family, PLDα1/2 and PLDβ1/2, were increased under the functional deficiency of PLDζ2 (Li *et al.*, 2006).

Based on the similarity of *Arabidopsis* PLDζ2 to mammalian PLD2, specific mammalian PLD2 inhibitor (*N*‐{2‐[4‐oxo‐1‐phenyl‐1,3,8‐triazaspiro(4.5)decan‐8‐yl]ethyl}quinoline‐3‐carboxamide), which also specifically inhibits the activity of AtPLDζ and does not affect other PLD members (Scott *et al.*, 2009; Yao *et al.*, 2013), was applied. Analysis showed that treatment with the PLD2 inhibitor did not affect primary root length or root hair number under either NP or LP conditions (Fig. S6). However, the root hair length of inhibitor‐treated plants was much shorter than those of the control (DMSO‐treated) plants under LP conditions (Fig. 2d), which was similar to the phenotype of *pin2* plants under LP conditions. In addition, PLD2 inhibitor suppressed the accumulation of PIN2 proteins (Figs 2b (bottom), S3) and decreased the level of auxin in the root hair zone under LP conditions (Fig. S4), further confirming that PLDζ2 was involved in root development under P deficiency through regulating the PIN2‐mediated auxin transport.

Knockout of PLDζ2 did not disrupt the root hair development under LP condition, whereas treatment with PLDζ2 inhibitor did. The discrepancies between gene knockout and treatment with inhibitor may be due to the fact that genetic deficiency of *PLDζ2* is complemented by other PLD members, whereas inhibitor specifically inhibited the activity of PLDζ2 protein and other PLD members could not complement this.

### Regulation of PIN2 vacuolar accumulation by PLDζ2‐derived phosphatidic acid is dependent on SNX1

Since PIN2 is a membrane protein, its cycling from the plasma membrane to the cytosol and sorting into the vacuole are crucial for its accumulation and activity in the membrane; these processes are largely dependent on SNX1 (Kleine‐Vehn *et al.*, 2008). Under low or no‐sucrose conditions, *snx1* mutant seedlings exhibit multiple auxin‐related defects, as SNX1 controls the transport of PIN2 through SNX1‐containing endosomes (Jaillais *et al.*, 2016; Hirano *et al.*, 2015). To explore whether SNX1 is involved in PIN2‐regulated root hair elongation under P deficiency, we assessed root development in *snx1* plants. Under either NP or LP conditions, primary root, lateral root, and root hair development were similar in *snx1* and WT (Fig. S7), which perhaps may be due to the functional redundancy of SNX family members (Pourcher *et al.*, 2010).

**Figure 3:**
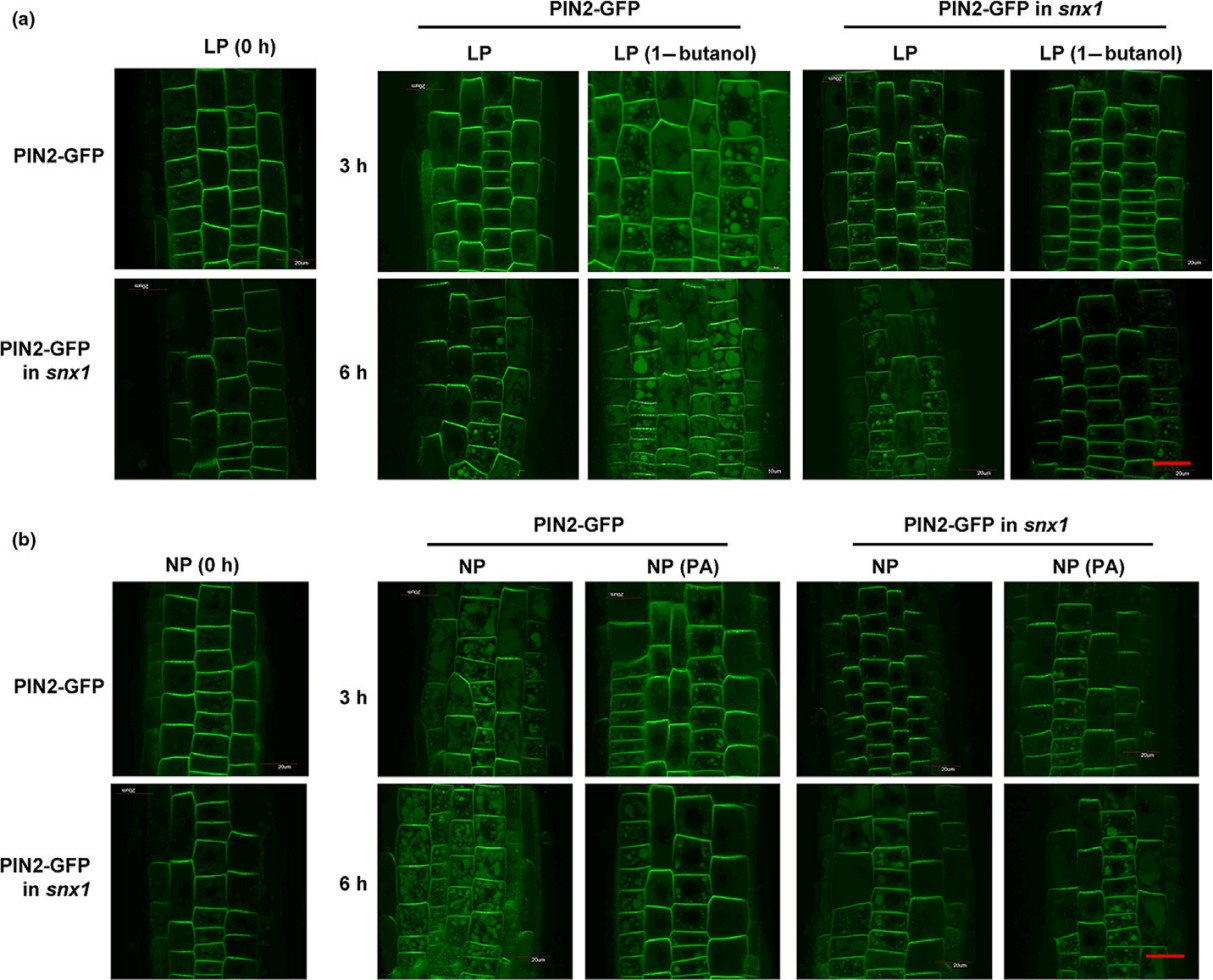
PIN‐FORMED2 (PIN2) degradation in vacuoles is dependent on SORTING NEXIN1 (SNX1). (a) PIN2 accumulation in vacuoles under low phosphate (LP) conditions (0 mM P_i_) in response to 1‐butanol treatment. Fluorescence of the PIN2‐green fluorescent protein (GFP) fusion protein was observed in the roots of *Arabidopsis thaliana* wild‐type (WT) or *snx1* plants under LP conditions; representative images are shown. Bar, 20 μm. (b) Phosphatidic acid (PA)‐suppressed PIN2 protein accumulation in vacuoles under normal phosphate (NP) conditions (1.25 mM P_i_) is reduced in the *snx1* background. Fluorescence of PIN2‐GFP fusion protein was observed in the roots of WT and *snx1* plants under NP conditions; representative images are shown. Bar, 20 μm.

Treatment with wortmannin (an inhibitor of phosphoinositide 3‐kinase), which interferes with the action of SNX under LP or NP conditions, resulted in shorter root hairs. In particular, the elongation of root hairs under LP conditions was significantly suppressed by wortmannin (Fig. 2d). Studies have shown that SNX1 plays an important role in regulating the auxin distribution in roots. Interestingly, under LP conditions, treatment with wortmannin results in an increased auxin level in root tips, while that at root elongation zones where root hair initiates was obviously decreased (Fig. S8), suggesting the involvement of SNXs in root hair elongation through regulating the distribution of auxin in root elongation zones. PIN2 accumulation at the plasma membrane is reduced in *snx1* seedlings (Kleine‐Vehn *et al.*, 2008), and further investigations on the recycling of PIN2 under LP conditions showed that compared to control, the PIN2 vacuolar accumulation under LP conditions was increased in response to treatment with 1‐butanol in WT, or that under NP conditions was reduced by the addition of exogenous PA (Fig. 2c), however, these were not observed in *snx1* seedlings (Fig. 3a,b), indicating that PLDζ2‐PA‐regulated PIN2 recycling under LP conditions was dependent on SNX1.

### Phosphatidic acid binds to SNX1 *in vitro* and promotes its accumulation at the plasma membrane under low phosphate condition

**Figure 4:**
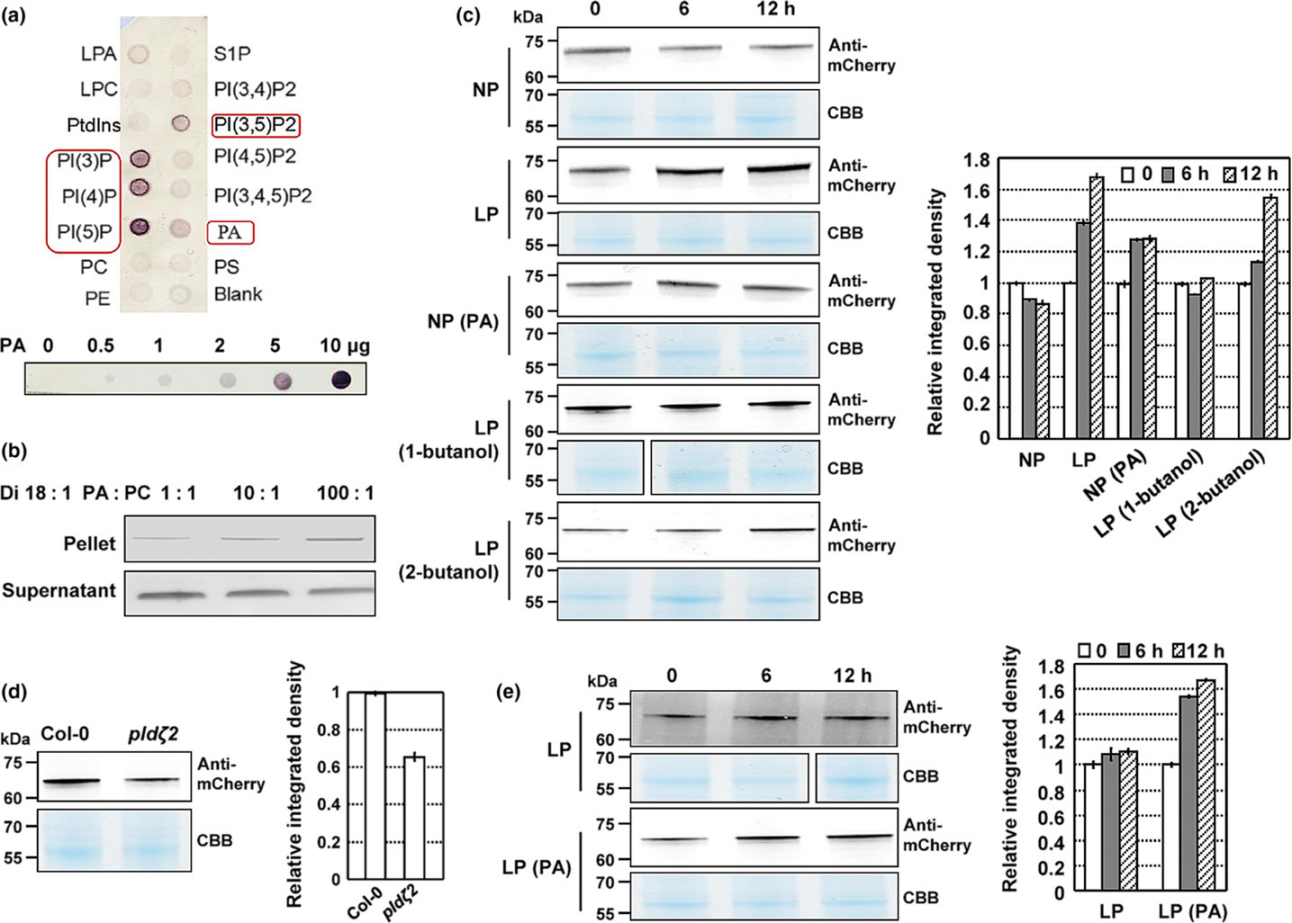
Phosphatidic acid (PA) binds to SORTING NEXIN1 (SNX1) and promotes its accumulation at the plasma membrane under low phosphate (LP) conditions. (a) Fat‐immunoblotting assay revealing the binding of SNX1 to PA. The phospholipid type of each dot is indicated (top). PC, phosphatidylcholine; PE, phosphatidylethanolamine; PS, phosphatidylserine; LPA, lysophosphatidic acid; LPC, lysophosphatidylcholine; S1P, sphingosine 1‐phosphate; PtdIns, phosphatidylinositol; PI(3)P, phosphatidylinositol 3‐phosphate; PI(4)P, phosphatidylinositol 4‐phosphate; PI(5)P, phosphatidylinositol 5‐phosphate; PI(3,4)P2, phosphatidylinositol 3,4‐phosphate; PI(3,5)P2, phosphatidylinositol 3,5‐phosphate; PI(4,5)P2, phosphatidylinositol 4,5‐phosphate; PI(3,4,5)P3, phosphatidylinositol 3,4,5‐phosphate. Different concentrations of PA (0, 0.5, 1, 2, 5 and 10 μg) were analyzed (bottom). (b) Liposome binding assay showing the correlation between bound SNX1 and PA. Purified histidine‐tagged SNX1 was incubated with liposomes containing different ratios of PC and PA. Bound (pellet with liposomes) SNX1 was detected by immunoblotting. Nonbinding protein was detected in the supernatant (bottom). (c–e) Immunoblot analysis of (c) AtSNX1‐mCherry protein abundance in *Arabidopsis thaliana* Col‐0 plants under normal phosphate (NP) (1.25 mM P_i_) or LP conditions (0 mM P_i_), under NP conditions with PA treatment (50 μM), or under LP conditions with 0.2% 1‐butanol or 0.2% 2‐butanol treatment; (d) Col‐0 or *pldζ2* plants under NP conditions; (e) *pldζ2* plants under LP conditions or under LP conditions with PA treatment (50 μM). Seven‐day‐old Col‐0 or *pldζ2* plants expressing AtSNX1‐mCherry were used to extract the plasma membrane proteins, which were examined by immunoblot analysis using anti‐mCherry antibody. Band density was measured using ImageJ, and data are presented as means ± SEM (*n* = 3). CBB, Coomassie Brilliant Blue.

Like human SNX1, *Arabidopsis* SNX1 contains a lipid‐binding domain (Phox homology (PX)), which binds to phosphatidylinositol 3‐phosphate (PI3P) and phosphatidylinositol 3,5‐bisphosphate (PI(3,5)P_2_) *in vitro* (Hirano *et al.*, 2015). To explore how PLDζ2‐PA regulates SNX1 activity, we examined the binding of purified SNX1 with lipids *in vitro* by performing a protein‐lipid overlay assay. In addition to PI3P, phosphatidylinositol 4‐phosphate, phosphatidylinositol 5‐phosphate, and PI(3,5)P_2_, SNX1 also interacted with PA in a dose‐dependent manner (Fig. 4a). A liposomal binding assay was further performed to verify the SNX1‐PA binding. The liposomes were produced with a mixture of dioleoyl PC and dioleoyl PA, and results confirmed the specific SNX1‐PA binding (Fig. 4b).

Therefore, we examined the cellular effects of PA binding with SNX1 and determined whether PA affects the accumulation of SNX1 in the membrane under LP conditions. We extracted membrane proteins from *Arabidopsis* seedlings expressing *SNX1*‐mCherry and analyzed the subcellular distribution of SNX1. The SNX1 accumulation at the membrane was increased under LP conditions, but treatment with 1‐butanol prevented this increase from occurring (Fig. 4c). Under NP conditions, SNX1 accumulated markedly at the plasma membrane upon PA treatment (Fig. 4c). Conversely, much less SNX1 was accumulated at the plasma membrane under NP conditions in *pld*ζ2 compared with WT (Fig. 4d). However, SNX1 accumulation at the plasma membrane of *pld*ζ2 seedlings was not obviously altered under LP conditions and was promoted by exogenous PA treatment (Fig. 4e). These results suggest that PA promoted SNX1 accumulation at the plasma membrane under LP conditions.

### The endocytosis and exocytosis of SNXs are unaltered, whereas PIN2 endocytosis is reduced under low phosphate conditions

**Figure 5:**
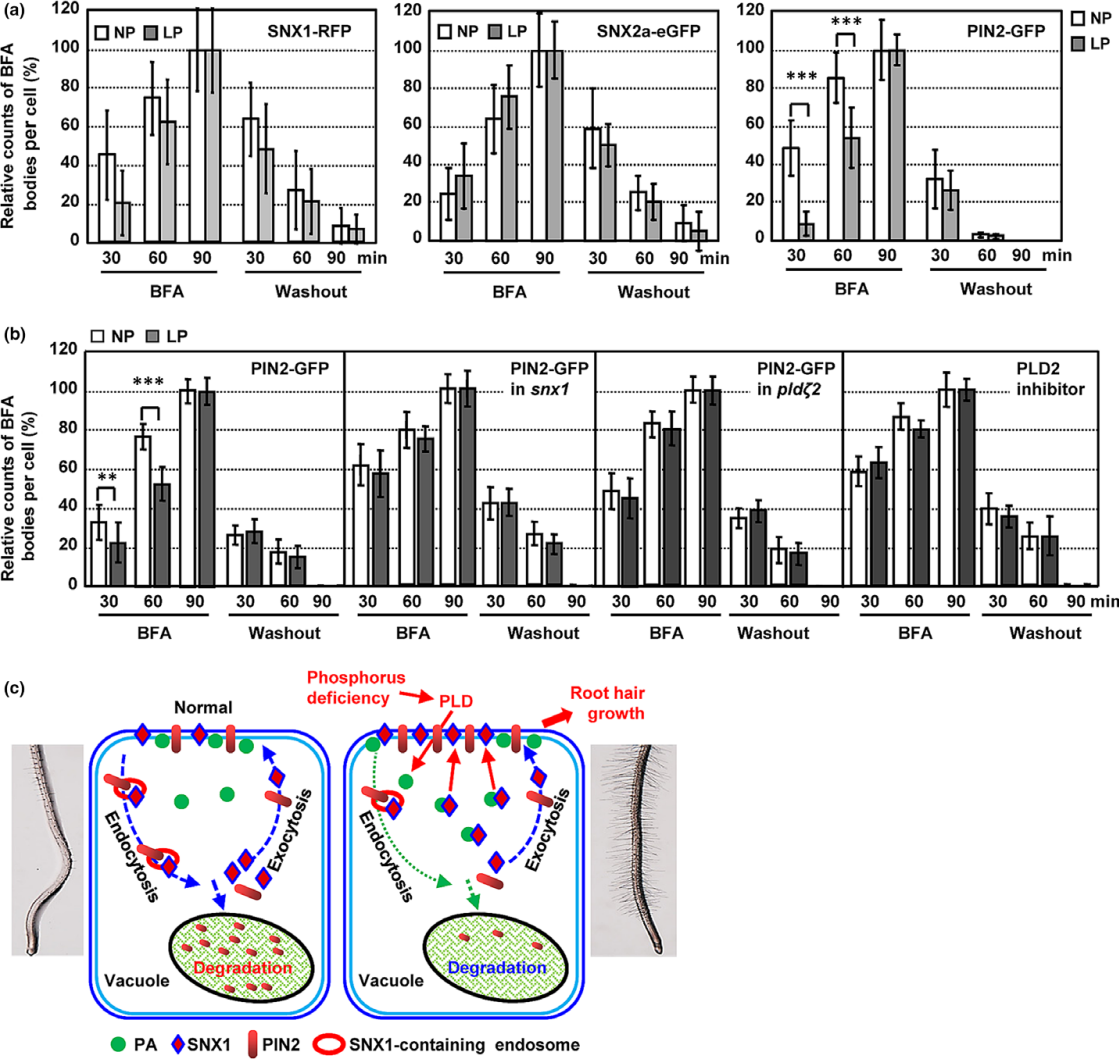
Low phosphate (LP) conditions suppress endocytosis but not exocytosis of PIN‐FORMED2 (PIN2) and do not affect either process for SORTING NEXIN (SNX)1 or SNX2a. (a) Number of Brefeldin A (BFA) bodies labeled with SNX1‐red fluorescent protein (RFP), SNX2a‐enhanced green fluorescent protein (eGFP), and PIN2‐green fluorescent protein (GFP). Five‐day‐old wild‐type (WT) *Arabidopsis thaliana* seedlings expressing PIN2‐GFP, pSNX1:SNX1‐RFP or pSNX2a:SNX2a‐eGFP were treated with BFA (45 μM) for 0, 30, 60 or 90 min, followed by washout with ½ Murashige & Skoog (½MS) for 30, 60 or 90 min under both normal phosphate (NP; 1.25 mM P_i_) and LP conditions (0 mM P_i_). Relative compartment counts per cell were calculated by setting the number of BFA bodies after treatment for 90 min as ‘100%’. Data are presented as means ± SE (*n *> 40) and statistically analyzed using a two‐tailed Student's *t*‐test (***, *P* < 0.001, compared with those under NP conditions at same time treatment). (b) Number of BFA bodies labeled PIN2‐GFP. Five‐day‐old WT seedlings expressing PIN2‐GFP in *snx1* mutant, in *pldζ2* mutant, or treated with PLD2 inhibitor (300 nM) were treated with BFA (45 μM) for 0, 30, 60 or 90 min, followed by washout with ½MS for 30, 60 or 90 min under both NP or LP conditions. Relative compartment counts per cell were calculated by setting the number of BFA bodies after treatment for 90 min as ‘100%’. Data are presented as means ± SE (*n* > 40) and statistically analyzed using a two‐tailed Student's *t*‐test (**, *P* < 0.01; ***, *P* < 0.001, compared with those under NP conditions at same time treatment). (c) Hypothetical model illustrating how PLDζ2‐derivated phosphatidic acid (PA) suppresses the degradation of PIN2 in the vacuole and stimulates root hair growth under LP conditions by binding with and promoting the membrane distribution of SNX1. Under NP conditions, the membrane protein PIN2 cycles from the membrane to the cytosol, and some of it is sorted into the vacuole for degradation directed by SNX‐containing endosomes (left). LP conditions stimulate PLDζ2 activity, thereby increasing PA levels, which binds to SNX1 and promotes the plasma membrane accumulation of SNX1, resulting in the suppressed endocytosis and the increased accumulation at the plasma membrane of PIN2, in turn promoting root hair growth under LP conditions (right).

In addition to regulating the subcellular localization of its binding proteins, PA also enhances the endocytosis of membrane proteins (Scott *et al.*, 2009). Since SNX1 protein is accumulated at the plasma membrane under LP conditions, we examined the endocytosis and exocytosis processes of both SNX1 and SNX2a using Brefeldin A, an inhibitor of ARF‐GEF‐type vesicle transport, by observing the aggregated endosomes (BFA bodies; Jaillais *et al.*, 2016). Observation and counting of BFA bodies under BFA treatment or after BFA washout revealed the similar levels of endocytosis and exocytosis of SNX1 and SNX2a under NP and LP conditions (Figs 5a, S9a,b), suggesting that the LP condition does not affect the endocytosis and exocytosis of the SNX retromer complex, and altered SNX1 accumulation at the plasma membrane is mainly due to the binding with PA not the vesicle trafficking.

Intercellular trafficking is important for PIN2 cycling and vacuolar degradation, and SNX1, a component of the retromer complex, resides in intracellular structures to function in the cycling of PIN2 (Jaillais *et al.*, 2016). Since SNX1 is accumulated at the plasma membrane under LP conditions, we thus investigated the endocytosis and exocytosis of PIN2 under LP conditions. Compared with the rapidly assembled BFA bodies under NP conditions, much fewer and smaller BFA bodies were observed under LP conditions (Figs 5a, S9c), indicating that the endocytosis of PIN2 was decreased under LP conditions. By contrast, after BFA washout, the number and sizes of BFA bodies harboring PIN2 were similar under NP and LP conditions, indicating that the exocytosis of PIN2 was unchanged under LP conditions. These results indicated that the reduced endocytosis of PIN2 under LP conditions was caused by the accumulated SNX1 at the plasma membrane. In addition, compared with that under NP conditions, both endocytosis and exocytosis of PIN2 under LP conditions were unaltered in *pldζ2* and *snx1* mutant or under treatment with PLD2 inhibitor (Figs 5b, S10), further confirming that vacuolar degradation of PIN2 under LP conditions was dependent on SNX1 and PLDζ2 and again suggesting that the reduced internalization of PIN2 through SNX1 and PLDζ2 under LP conditions led to the suppressed vacuolar accumulation of PIN2.

## Discussion

In this study, we demonstrated the crucial roles of the auxin efflux carrier PIN2 in plant responses to P deficiency through regulating root hair elongation. The degradation and intercellular trafficking of PIN2 are suppressed by PLD‐derived PA through its binding with SNX1. Since PLDs are significantly induced by P deficiency, our results reveal a regulatory mechanism of root hair elongation under P deficiency via PLD‐derived PA, which suppresses the vacuolar degradation of PIN2 by binding to SNX1 (Fig. 5c).

### The crucial role of PIN2 in root hair elongation under phosphorus deficiency

Root is the main organ for nutrient uptake, and root system architecture is significantly altered under nutrient deficiency, especially P deficiency. Root hair number and elongation are affected under LP conditions, which improves the absorption capacity and efficiency of roots (Bates & Lynch, 2000; Ticconi *et al.*, 2004).

Auxin is critical for root development, and its abundance increases in the root tip and maturation zone (where root hair initiates) under LP conditions (Nacry *et al.*, 2005; Bhosale *et al.*, 2018), indicating that IAA accumulation in the maturation zone is necessary for the development of root hair (Bhosale *et al.*, 2018). Moreover, root hair development and elongation are affected by polar auxin transport, and P deficiency mediates the polarization of PIN2 by affecting the levels of strigolactones (Jone *et al.*, 2009; Kumar *et al.*, 2015). PIN family genes are transcribed in *Arabidopsis* root hair cells, and *PIN2* is expressed at higher levels than other PIN genes (Lee & Cho, 2006). The specific expression of *PIN2* in root hair cells results in decreased internal auxin levels in these cells, greatly inhibiting root hair growth (Ganguly *et al.*, 2010). Indeed, PIN2 deficiency results in suppressed auxin transport from root tips to the elongation zone, leading to the accumulation of auxin at the root tip and thus to defective root hair elongation (Liu *et al.*, 2018). We found that the internalization and degradation of PIN2 in vacuoles is reduced under LP conditions. The accumulated PIN2 promotes auxin transport from the root tip to the root hair zone and increases the auxin levels of root hair cells, resulting in increased root hair elongation under LP conditions. In addition, both influx carriers (AUX1) and *PIN2* are expressed in the epidermis cells, and *aux1* mutant presents defective root hair development under LP conditions, which can be rescued when AUX1 is expressed in lateral root cap and epidermal cells (Bhosale *et al.*, 2018). Considering that the defective root hair phenotype of *pin2* is similar to that of *aux1*, it is suggested that the accumulated PIN2 in the epidermal cells of root tip under LP conditions leads to the increased auxin level in the root hair zone, hence promoting the development of root hair, like AUX1. In addition, although no evidence showing that mutants of other PIN transporters (PIN1, PIN3) present abnormal root hair growth, whether they are involved in the root hair growth regulation, particularly under P deficiency, is unknown and needs further investigations.

### Phosphatidic acid or phosphorus deficiency suppresses vacuolar degradation of PIN2

In addition to transcriptional regulation, PIN2 exhibits multiple levels of posttranslational regulation, including phosphorylation and ubiquitination (Michniewicz *et al.*, 2007; Leitner *et al.*, 2012). We demonstrated that PIN2 dynamically accumulates and is degraded in the vacuole under NP conditions. Interestingly, the vacuolar degradation of PIN2 is suppressed under LP conditions, which is consistent with the enhanced PIN2 protein levels in root tips and the suppressed response of *pin2* to P deficiency.

PLD‐derived PA plays crucial roles in plant responses to various environmental stresses, such as salinity and P deficiency (Li *et al.*, 2006; Wang *et al.*, 2019). PA interacts with protein kinase PID and increases the PID‐dependent phosphorylation of PIN2, which enhances the auxin efflux activity and PIN2 accumulation at the plasma membrane under salt stress (Wang *et al.*, 2019). Our results demonstrated that PLDζ2‐derived PA that is induced by P deficiency mediates the vacuolar degradation of PIN2 to increase the plant response to low P tolerance. Considering the multiple members of the PLD family, various environmental stresses may utilize different mechanisms, mediated by a distinct PLD member or PA species, to regulate the auxin levels through PIN2.

P_i_ availability alters lipid composition and content in plant tissues (Cruz‐Ramírez *et al.*, 2006; Li *et al.*, 2006). Although PLD family members, especially PLDζ2, are significantly induced under P deficiency, how PLDζ2 is involved in plant responses to P deficiency is currently unknown. Overexpression of PLDζ2 or treatment with PA increases auxin levels in roots, whereas the *pldζ2* mutation or treatment with 1‐butanol decreases auxin levels (Li & Xue 2007). Interestingly, both PIN2 and PLDζ2 are expressed in the epidermal cells of root tip (Taniguchi *et al.*, 2010). Enhanced vacuolar degradation of PIN2 in *pldζ2* under LP conditions and suppressed vacuolar degradation of PIN2 under NP conditions in response to PA were detected, which is consistent with the decreased auxin level in root hair zones of *pldζ2* mutant under LP conditions. These observations are consistent with the notion that increased auxin levels during root elongation and increased root hair elongation under LP conditions are mediated by increased PA levels. These findings shed light on the involvement of the PLDζ2‐derived PA in plant responses to P deficiency through regulating auxin activity and root hair development, providing important clues about the regulatory network of plant responses to nutrient deficiency. It is noticed that the *pldζ2* mutant does not present developmental defects of roots under NP or LP conditions. Considering the difference with the reported phenotype of *pldζ2*, including the primary root and root hair (Cruz‐Ramírez *et al.*, 2006; Li *et al.*, 2006; Yao *et al.*, 2013; Su *et al.*, 2018), it is speculated that, based on the crucial roles of PLDs and PA in plant responses to the environment, one possible reason for the difference may due to the very slight difference in treatment conditions leading to the change in growth and development.

The PLD2 inhibitor specifically inhibits the activity of PLDζ but not other PLD members (Yao *et al.*, 2013). Although functional deficiency of PLDζ2 results in the increased transcriptions of some members of the PLD family (Li *et al.*, 2006), the unaltered transcriptions of them under PLD2 inhibitor do lead to the defective root hair development, confirming a specific role of PLDζ2 in regulating root hair growth under P deficiency.

### Phophatidic acid promotes accumulation of SNX1 at the plasma membrane through binding

PA binds to target proteins to regulate their activity or subcellular localization, thereby affecting various signaling or physiological processes (Yao & Xue, 2018). Our results demonstrate that PA binds to SNX1 to promote its distribution at the plasma membrane under LP conditions, thereby suppressing endocytosis and resulting in the accumulation of PIN2. *Arabidopsis* SNX1 contains two domains: the PX domain and the Bin/amphiphysin/Rvs domain. The PX domain contains phospholipid‐binding motifs. Human SNX1 specifically interacts with PI(3,5)P_2_ and phosphatidylinositol (3,4,5)‐trisphosphate, and *Arabidopsis* SNX1 directly binds to PI3P and PI(3,5)P_2_ *in vitro* (Zhong *et al.*, 2002; Hirano *et al.*, 2015). In *snx1* mutant or treatment with wortmannin, the auxin transport and distribution are disturbed, which is similar to the effects of 1‐butanol treatment (Li & Xue 2007; Jaillais *et al.*, 2016), pointing to a relationship between SNX1 and PLD‐PA. Our results demonstrated that SNX1 also binds with PA and that PA‐regulated vacuolar degradation of PIN2 is dependent on SNX1, shedding light on the roles and mechanism of phospholipids in plant responses to nutrient deficiency.

PA stimulates the endocytosis of membrane proteins. However, cytological analysis revealed that PA does not alter the vesicle transport of SNX1 under LP conditions, indicating that PA stimulates the plasma membrane distribution of SNX1 under LP conditions primarily via direct binding. Although similarly suppressed vacuolar degradation of PIN2 was observed under increased PA and IP_3_ levels, IP_3_ specially promotes SNX1 endocytosis, resulting in reduced recruitment to and accumulation of SNX1 at the plasma membrane (Chu *et al.*, 2016). These findings indicate that plants employ different regulatory mechanisms in response to distinct environmental factors.

Phosphatidylinositol signaling and SNXs are highly conserved in mammalian and plant cells, and SNXs play crucial roles in regulating lysosomal trafficking and protein degradation in mammalian cells. As a key signaling molecule in both mammals and plants, therefore, perhaps PA plays a role in regulating the degradation of membrane proteins in lysosomes by modulating SNX distribution, representing a general regulatory mechanism throughout eukaryotic species.

## Author contributions

D‐LL performed acquisition of data as well as analysis and interpretation of data and drafted the manuscript. H‐YY performed the fat Western analysis and drafted the manuscript. L‐HJ helped in preparing the seeds, J‐FT and Z‐HX helped with design and discussions. W‐MZ and H‐WX were responsible for conception and design, as well as analysis and interpretation of data and revised the article. D‐LL and H‐YY contributed equally to this work.

## Supporting information

Please note: Wiley Blackwell are not responsible for the content or functionality of any Supporting Information supplied by the authors. Any queries (other than missing material) should be directed to the *New Phytologist* Central Office.

**Fig. S1** Primary and lateral root growth is not altered in *pin2* under LP condition.
**Fig. S2** Visualization of accumulation of PIN2‐GFP protein to the vacuole.
**Fig. S3** PIN2 protein is accumulated under LP condition.
**Fig. S4** Increased auxin level at root tips and elongation zones under LP condition.
**Fig. S5** Primary and lateral roots and root hairs are not altered in *pldζ2* and PLDζ2ox under LP condition.
**Fig. S6** Treatment with PLD2 inhibitor does not alter the primary root length or root hair number.
**Fig. S7** Primary and lateral roots and root hairs are not altered in *snx1* under LP condition.
**Fig. S8** Altered auxin levels at root elongation zones under LP condition with Wortmannin treatment.
**Fig. S9** LP condition suppresses the endocytosis but not the exocytosis of PIN2 and does not affect the endocytosis or exocytosis of SNX1 or SNX2a.
**Fig. S10** Unaltered endocytosis of PIN2 under LP condition or *PLDζ2 *and SNX1 deficiency.

## References

[nph16330-bib-0002] Andersson MX , Stridh MH , Larsson KE , Liljenberg C , Sandelius AS . 2003 Phosphate‐deficient oat replaces a major portion of the plasma membrane phospholipids with the galactolipid digalactosyldiacylglycerol. FEBS Letters 537: 128–132. 12606044 10.1016/s0014-5793(03)00109-1

[nph16330-bib-0004] Bates TR , Lynch JP . 2000 The efficiency of *Arabidopsis thaliana* (Brassicaceae) root hairs in phosphorus acquisition. American Journal of Botany 87: 964–970. 10898773

[nph16330-bib-0005] Bhosale R , Giri J , Pandey BK , Giehl RFH , Hartmann A , Traini R , Truskina J , Leftley N , Hanlon M , Swarup K *et al* 2018 A mechanistic framework for auxin dependent *Arabidopsis* root hair elongation to low external phosphate. Nature Communications 9: e1409. 10.1038/s41467-018-03851-3 PMC5897496 29651114

[nph16330-bib-0006] Chu YJ , Chen X , Xue HW . 2016 Ins(1,4,5)P_3_ suppresses protein degradation in plant vacuoles by regulating SNX‐mediated protein sorting. Molecular Plant 9: 1440–1443. 27477682 10.1016/j.molp.2016.07.009

[nph16330-bib-0007] Cruz‐Ramírez A , Oropeza‐Aburto A , Razo‐Hernández F , Ramirez‐Chávez E , Herrera‐Estrella L . 2006 Phospholipase DZ2 plays an important role in extraplastidic galactolipid biosynthesis and phosphate recycling in *Arabidopsis* roots. Proceedings of the National Academy of Sciences, USA 103: 6765–6770. 10.1073/pnas.0600863103 PMC1564204 16617110

[nph16330-bib-0008] Cui YY , Li XG , Chen QG , He X , Yang Q , Zhang AL , Yu X , Chen H , Liu NY , Xie Q *et al* 2010 BLOS1, a putative BLOC‐1 subunit, interacts with SNX1 and modulates root growth in *Arabidopsis* . Journal of Cell Science 123: 3727–3733. 20971704 10.1242/jcs.069732

[nph16330-bib-0009] Friml J , Wiśniewska J , Benková E , Mendgen K , Palme K . 2002 Lateral relocation of auxin efflux regulator PIN3 mediates tropism in *Arabidopsis* . Nature 415: 806–809. 11845211 10.1038/415806a

[nph16330-bib-0010] Ganguly A , Lee SH , Cho M , Lee OR , Yoo H , Cho HT . 2010 Differential auxin‐transporting activities of PIN‐FORMED proteins in *Arabidopsis* root hair cells. Plant Physiology 153: 1046–1061. 20439545 10.1104/pp.110.156505 PMC2899906

[nph16330-bib-0011] Gao HB , Chu YJ , Xue HW . 2013 Phosphatidic acid (PA) binds PP2AA1 to regulate PP2A activity and PIN1 polar localization. Molecular Plant 6: 1692–1702. 23686948 10.1093/mp/sst076

[nph16330-bib-0012] Giri J , Bhosale R , Huang G , Pandey BK , Parker H , Zappala S , Yang J , Dievart A , Bureau C , Ljung K *et al* 2018 Rice auxin influx carrier *OsAUX1* facilitates root hair elongation in response to low external phosphate. Nature Communications 9: e1408. 10.1038/s41467-018-03850-4 PMC5897452 29650967

[nph16330-bib-0013] Hanzawa T , Shibasaki K , Numata T , Kawamura Y , Gaude T , Rahman A . 2013 Cellular auxin homeostasis under high temperature is regulated through a sorting NEXIN1‐dependent endosomal trafficking pathway. Plant Cell 25: 3424–3433. 24003052 10.1105/tpc.113.115881 PMC3809541

[nph16330-bib-0014] Heucken N , Ivanov R . 2018 The retromer, sorting nexins and the plant endomembrane protein trafficking. Journal of Cell Science 131: jcs203695. 29061884 10.1242/jcs.203695

[nph16330-bib-0015] Hirano T , Munnik T , Sato MH . 2015 Phosphatidylinositol 3‐phosphate 5‐kinase, FAB1/PIKfyve kinase mediates endosome maturation to establish endosome–cortical microtubule interaction in *Arabidopsis* . Plant Physiology 169: 1961–1974. 26353760 10.1104/pp.15.01368 PMC4634102

[nph16330-bib-0016] Ivanov R , Brumbarova T , Blum A , Jantke A , Fink‐Straube C , Bauer P . 2014 SORTING NEXIN1 is required for modulating the trafficking and stability of the *Arabidopsis* IRON‐REGULATED TRANSPORTER1. Plant Cell 26: 1294–1307. 24596241 10.1105/tpc.113.116244 PMC4001385

[nph16330-bib-0017] Jaillais Y , Fobis‐Loisy I , Miège C , Rollin C , Gaude T . 2016 AtSNX1 defines an endosome for auxin‐carrier trafficking in *Arabidopsis* . Nature 443: 106–109. 10.1038/nature05046 16936718

[nph16330-bib-0018] Jones AR , Kramer EM , Knox K , Swarup R , Bennett MJ , Lazarus CM , Leyser HMO , Grierson CS . 2009 Auxin transport through non‐hair cells sustains root‐hair development. Nature Cell Biology 11: 78–84. 19079245 10.1038/ncb1815 PMC2635559

[nph16330-bib-0019] Kapulnik Y , Resnick N , Mayzlish‐Gati E , Kaplan Y , Wininger S , Hershenhorn J , Koltai H . 2011 Strigolactones interact with ethylene and auxin in regulating root‐hair elongation in *Arabidopsis* . Journal of Experimental Botany 62: 2915–2924. 21307387 10.1093/jxb/erq464

[nph16330-bib-0020] Kleine‐Vehn J , Leitner J , Zwiewka M , Sauer M , Abas L , Luschnig C , Friml J . 2008 Differential degradation of PIN2 auxin efflux carrier by retromer‐dependent vacuolar targeting. Proceedings of the National Academy of Sciences, USA 105: 17812–17817. 10.1073/pnas.0808073105 PMC2584678 19004783

[nph16330-bib-0021] Kumar M , Pandya‐Kumar N , Dam A , Haor H , Mayzlish‐Gati E , Belausov E , Wininger S , Abu‐Abied M , McErlean CSP , Brombead LJ *et al* 2015 *Arabidopsis* response to low‐phosphate condition includes active changes in actin filaments and PIN2 polarization and is dependent on strigolactone signalling. Journal of Experimental Botany 66: 1499–1510. 25609825 10.1093/jxb/eru513 PMC4339606

[nph16330-bib-0022] Kurten RC , Cadena DL , Gill GN . 1996 Enhanced degradation of EGF receptors by a sorting nexin, SNX1. Science 272: 1008–1010. 8638121 10.1126/science.272.5264.1008

[nph16330-bib-0023] Laxmi A , Pan J , Morsy M , Chen R . 2008 Light plays an essential role in intracellular distribution of auxin efflux carrier PIN2 in *Arabidopsis thaliana* . PLoS ONE 3: e1510. 18231596 10.1371/journal.pone.0001510 PMC2200863

[nph16330-bib-0024] Lee SH , Cho HT . 2006 PINOID positively regulates auxin efflux in *Arabidopsis* root hair cells and tobacco cells. Plant Cell 18: 1604–1616. 16731587 10.1105/tpc.105.035972 PMC1488908

[nph16330-bib-0025] Leitner J , Retzer K , Korbei B , Luschnig C . 2012 Dynamics in PIN2 auxin carrier ubiquitylation in gravity‐responding *Arabidopsis* roots. Plant Signaling Behavior 7: 1271–1273. 22902683 10.4161/psb.21715 PMC3493411

[nph16330-bib-0026] Li G , Xue HW . 2007 *Arabidopsis* PLDζ2 regulates vesicle trafficking and is required for auxin response. Plant Cell 19: 281–295. 17259265 10.1105/tpc.106.041426 PMC1820954

[nph16330-bib-0027] Li M , Qin C , Welti R , Wang X . 2006 Double knockouts of phospholipases Dζ1 and Dζ2 in *Arabidopsis* affect root elongation during phosphate‐limited growth but do not affect root hair patterning. Plant Physiology 140: 761–770. 16384909 10.1104/pp.105.070995 PMC1361341

[nph16330-bib-0028] Li TT , Liu WC , Wang FF , Ma QB , Lu YT , Yuan TT . 2018 SORTING NEXIN 1 functions in plant salt stress tolerance through changes of NO accumulation by regulating NO synthase‐like activity. Frontiers in Plant Science 9: e1643. 10.3389/fpls.2018.01634 PMC6277890 30542353

[nph16330-bib-0029] Liu HB , Liu B , Chen XL , Zhu H , Zou CX , Men SZ . 2018 AUX1 acts upstream of PIN2 in regulating root gravitropism. Biochemical and Biophysical Research Communications 507: 433–436. 30449597 10.1016/j.bbrc.2018.11.056

[nph16330-bib-0030] López‐Bucio J , Hernández‐Abreu E , Sánchez‐Calderón L , Nieto‐Jacobo MF , Simpson J , Herrera‐Estrella L . 2002 Phosphate availability alters architecture and causes changes in hormone sensitivity in the *Arabidopsis* root system. Plant Physiology 129: 244–256. 12011355 10.1104/pp.010934 PMC155888

[nph16330-bib-0031] Martín‐Rejano EM , Camacho‐Critóbal JJ , Herrera‐Rodríguez MB , Rexach J , Navarro‐Gochicoa MT , González‐Fontes A . 2011 Auxin and ethylene are involved in the responses of root system architecture to low boron supply in Arabidopsis seedlings. Physiologia Plantarum 142: 170–178. 21338369 10.1111/j.1399-3054.2011.01459.x

[nph16330-bib-0032] Michniewicz M , Zago MK , Abas L , Weijers D , Schweighofer A , Meskiene I , Heisler MG , Ohno C , Zhang J , Huang F *et al* 2007 Antagonistic regulation of PIN phosphorylation by PP2A and PINOID directs auxin flux. Cell 130: 1044–1056. 17889649 10.1016/j.cell.2007.07.033

[nph16330-bib-0033] Munnik T , Arisz SA , De Vrije T , Musgrave A . 1995 G protein activation stimulates phospholipase D signaling in plants. Plant Cell 7: 2197–2210. 12242371 10.1105/tpc.7.12.2197 PMC161073

[nph16330-bib-0034] Nacry P , Canivenc G , Muller B , Azmi A , Onckelen HV , Rossignol M , Doumas P . 2005 A role for auxin redistribution in the responses of the root system architecture to phosphate starvation in *Arabidopsis* . Plant Physiology 138: 2061–2074. 16040660 10.1104/pp.105.060061 PMC1183395

[nph16330-bib-0035] Nakamura Y . 2013 Phosphate starvation and membrane lipid remodeling in seed plants. Progress in Lipid Research 52: 43–50. 22954597 10.1016/j.plipres.2012.07.002

[nph16330-bib-0036] Nakamura Y , Awai K , Masuda T , Yoshioka Y , Takamiya K , Ohta H . 2005 A novel phosphatidylcholine‐hydrolyzing phospholipase C induced by phosphate starvation in *Arabidopsis* . Journal of Biological Chemistry 280: 7469–7476. 15618226 10.1074/jbc.M408799200

[nph16330-bib-0037] Nakamura Y , Koizumi R , Shui G , Shimojima M , Wenk MR , Ito T , Ohta H . 2009 *Arabidopsis* lipids mediate eukaryotic pathway of lipid metabolism and cope critically with phosphate starvation. Proceedings of the National Academy of Sciences, USA 106: 20978–20983. 10.1073/pnas.0907173106 PMC2791602 19923426

[nph16330-bib-0038] Pant BD , Burgos A , Pant P , Cuadros‐Inostroza A , Willmitzer L , Scheible WR . 2015 The transcription factor PHR1 regulates lipid remodeling and triacylglycerol accumulation in *Arabidopsis thaliana* during phosphorus starvation. Journal of Experimental Botany 66: 1907–1918. 25680792 10.1093/jxb/eru535 PMC4378627

[nph16330-bib-0039] Péret B , Clément M , Nussaume L , Desnos T . 2011 Root developmental adaptation to phosphate starvation: better safe than sorry. Trends in Plant Science 16: 442–450. 21684794 10.1016/j.tplants.2011.05.006

[nph16330-bib-0040] Pérez‐Torres CA , López‐Bucio J , Cruz‐Ramírez A , Ibarra‐Laclette E , Dharmasiri S , Estelle M , Herrera‐Estrella L . 2008 Phosphate availability alters lateral root development in *Arabidopsis* by modulating auxin sensitivity via a mechanism involving the TIR1 auxin receptor. Plant Cell 20: 3258–3272. 19106375 10.1105/tpc.108.058719 PMC2630440

[nph16330-bib-0041] Petrasek J , Friml J . 2009 Auxin transport routes in plant development. Development 136: 2675–2688. 19633168 10.1242/dev.030353

[nph16330-bib-0042] Petrášek J , Mravec J , Bouchard R , Blakeslee JJ , Abas M , Seifertová D , Wiśniewska J , Tadele Z , Kubeš M , Čovanoá M *et al* 2006 PIN proteins perform a rate‐limiting function in cellular auxin. Science 312: 914–918. 16601150 10.1126/science.1123542

[nph16330-bib-0043] Plaxton WC , Tran HT . 2011 Metabolic adaptations of phosphate‐starved plants. Plant Physiology 156: 1006–1015. 21562330 10.1104/pp.111.175281 PMC3135920

[nph16330-bib-0044] Pourcher M , Santambrogio M , Thazar N , Thierry AM , Fobis‐Loisy I , Mièga C , Jaillais Y , Gaude T . 2010 Analyses of sorting nexins reveal distinct retromer‐subcomplex functions in development and protein sorting in *Arabidopsis thaliana* . Plant Cell 22: 3980–3991. 21156856 10.1105/tpc.110.078451 PMC3027177

[nph16330-bib-0045] Raghothama KG . 1999 Phosphate acquisition. Annual Review of Plant Biology 50: 665–693. 10.1146/annurev.arplant.50.1.665 15012223

[nph16330-bib-0046] Salanenka Y , Verstraeten I , Löfke C , Tabata K , Naramoto S , Glanc M , Friml J . 2018 Gibberellin DELLA signaling targets the retromer complex to redirect protein trafficking to the plasma membrane. Proceedings of the National Academy of Sciences, USA 115: 3716–3721. 10.1073/pnas.1721760115 PMC5889667 29463731

[nph16330-bib-0047] Sánchez‐Calderón L , López‐Bucio J , Chacón‐López A , Cruz‐Ramírez A , Nieto‐Jacobo F , Dubrovsky JG , Herrera‐Estrella L . 2005 Phosphate starvation induces a determinate developmental program in the roots of *Arabidopsis thaliana* . Plant Cell Physiology 46: 174–184. 15659445 10.1093/pcp/pci011

[nph16330-bib-0048] Seaman MNJ . 2005 Recycle your receptors with retromer. Trends in Cell Biology 15: 68–75. 15695093 10.1016/j.tcb.2004.12.004

[nph16330-bib-0049] Scott SA , Selvy PE , Buck JR , Cho HP , Criswell TL , Thomas AL , Armstrong MD , Arteaga CL , Lindsley CW , Brown HA . 2009 Design of isoform‐selective phospholipase D inhibitors that modulate cancer cell invasiveness. Nature Chemical Biology 5: 108–117. 19136975 10.1038/nchembio.140 PMC3798018

[nph16330-bib-0050] Shimojima M , Ohta H . 2011 Critical regulation of galactolipid synthesis controls membrane differentiation and remodeling in distinct plant organs and following environmental changes. Progress in Lipid Research 50: 258–266. 21414359 10.1016/j.plipres.2011.03.001

[nph16330-bib-0051] Su Y , Li M , Guo L , Wang X . 2018 Different effects of phospholipase Dζ2 and non‐specific phospholipase C4 on lipid remodeling and root hair growth in *Arabidopsis* response to phosphate deficiency. The Plant Journal 94: 315–326. 29437261 10.1111/tpj.13858

[nph16330-bib-0052] Tan ST , Dai C , Liu HT , Xue HW . 2013 *Arabidopsis* casein kinase1 proteins CK1.3 and CK1.4 phosphorylate cryptochrome2 to regulate blue light signaling. Plant Cell 25: 2618–2632. 23897926 10.1105/tpc.113.114322 PMC3753387

[nph16330-bib-0053] Tang Y , Zhao CY , Tan ST , Xue HW . 2016 *Arabidopsis* type II phosphatidylinositol 4‐kinase PI4Kγ5 regulates auxin biosynthesis and leaf margin development through interacting with membrane‐bound transcription factor ANAC078. PLoS Genetics 12: e1006252. 27529511 10.1371/journal.pgen.1006252 PMC4986951

[nph16330-bib-0054] Taniguchi YY , Taniguchi M , Tsuge T , Oka A , Aoyama T . 2010 Involvement of *Arabidopsis thaliana* phospholipase Dζ2 in root hydrotropism through the suppression of root gravitropism. Planta 231: 491–497. 19915862 10.1007/s00425-009-1052-x

[nph16330-bib-0055] Ticconi CA , Delatorre CA , Lahner B , Salt DE , Abel S . 2004 *Arabidopsis pdr2* reveals a phosphate‐sensitive checkpoint in root development. The Plant Journal 37: 801–814. 14996215 10.1111/j.1365-313x.2004.02005.x

[nph16330-bib-0056] Wang PP , Shen LK , Guo JH , Jing W , Qu YN , Li WY , Bi RR , Xuan W , Zhang Q , Zhang WH . 2019 Phosphatidic acid directly regulates PINOID‐dependent phosphorylation and activation of the PIN‐FORMED2 auxin efflux transporter in response to salt stress. Plant Cell 31: 250–271. 30464035 10.1105/tpc.18.00528 PMC6391703

[nph16330-bib-0057] Wiśniewska J , Xu J , Seifertová D , Brewer PB , Růžička K , Blilou I , Rouquié D , Benková E , Scheres B , Friml J . 2006 Polar PIN localization directs auxin flow in plants. Science 312: 883. 16601151 10.1126/science.1121356

[nph16330-bib-0059] Yao H , Wang G , Guo L , Wang X . 2013 Phosphatidic acid interacts with a MYB transcription factor and regulates its nuclear localization and function in *Arabidopsis* . Plant Cell 25: 5030–5042. 24368785 10.1105/tpc.113.120162 PMC3904003

[nph16330-bib-0058] Yao H , Xue HW . 2018 Phosphatidic acid plays key roles regulating plant development and stress responses. Journal of Integrative Plant Biology 60: 851–863. 29660254 10.1111/jipb.12655

[nph16330-bib-0060] Zhong Q , Lazar CS , Tronchère H , Sato T , Meerloo T , Yeo M , Zhou SY , Emr SD , Gill GN . 2002 Endosomal localization and function of sorting nexin 1. Proceedings of the National Academy of Sciences, USA 99: 6767–6772. 10.1073/pnas.092142699 PMC124477 11997453

