# Supplementary material for: Phospholipase D‐derived phosphatidic acid promotes root hair development under phosphorus deficiency by suppressing vacuolar degradation of PIN‐FORMED2
Source: New Phytol. 2019 Dec 19;226(1):142–55. doi: 10.1111/nph.16330 (PMC7065129; doi:10.1111/nph.16330)

New Phytologist Supporting Information

Article title: **Phospholipase D-derived phosphatidic acid promotes root hair development under phosphorus deficiency by suppressing vacuolar degradation of PIN2**

Authors: De-Li Lin, Hong-Yan Yao, Li-Hua Jia, Jin-Fang Tan, Zhi-Hong Xu, Wen-Ming Zheng, Hong-Wei Xue

Article acceptance date: 10 November 2019

Fig. S1 Primary and lateral root growth is not altered in *pin2* under LP condition.

Fig. S2 Visualization of accumulation of PIN2-GFP protein to the vacuole.

Fig. S3 PIN2 protein is accumulated under LP condition.

Fig. S4 Increased auxin level at root tips and elongation zones under LP condition.

Fig. S5 Primary and lateral roots and root hairs are not altered in *pldζ2* and PLDζ2ox under LP condition.

Fig. S6 Treatment with PLD2 inhibitor does not alter the primary root length or root hair number.

Fig. S7 Primary and lateral roots and root hairs are not altered in *snx1* under LP conditions.

Fig. S8 Altered auxin levels at root elongation zones under LP condition with Wortmannin treatment.

Fig. S9 LP condition suppresses the endocytosis but not the exocytosis of PIN2 and does not affect the endocytosis or exocytosis of SNX1 or SNX2a.

Fig. S10 Unaltered endocytosis of PIN2 under LP condition or *PLDζ2* and SNX1 deficiency.

Fig. S1. Primary and lateral root growth is not altered in *pin2* under LP condition. Col-0 and *pin2* seedlings were grown under NP (1.25 mM Pi) or LP (0 mM Pi) conditions for 3, 5, or 7 days, and primary root length (upper) and lateral root number (bottom) were measured. The experiments were performed with three biological repeats, and data are presented as means ± SE ($n > 50$). Statistical analysis by two-tailed Student's t-test revealed no difference compared to Col-0.

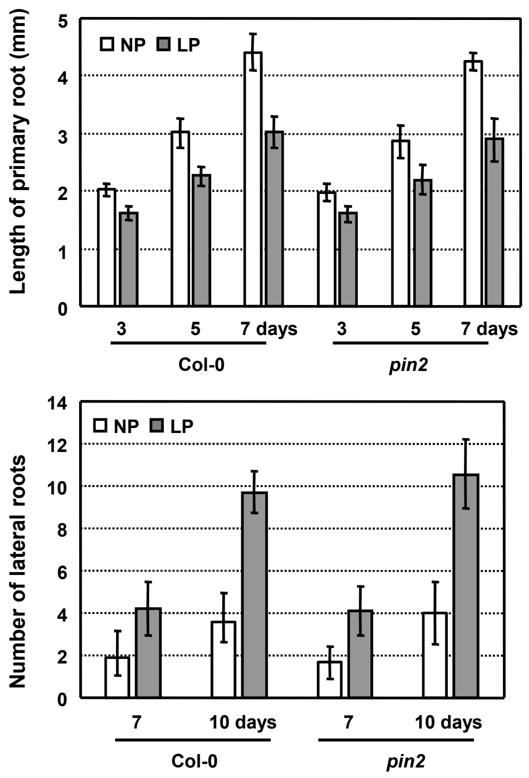

Fig. S2. Visualization of accumulation of PIN2-GFP protein to the vacuole. Seedling expressing PIN2-GFP were grown on 1/2 MS for 4 days and transferred to dark (3 or 6 h). GFP fluorescence in root cells was observed and FM4-64 was used to label the tonoplast around the diffuse GFP signal. Bar = 20 µm.

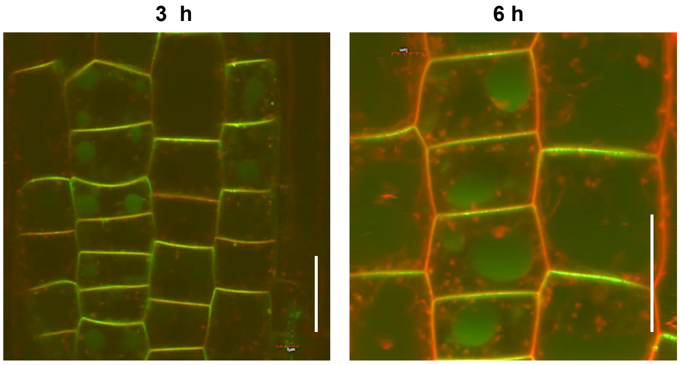

Fig. S3. PIN2 protein is accumulated under LP condition. Immunoblot analysis of PIN2-GFP protein abundance at root tips under NP (1.25 mM Pi) or LP conditions (a), or LP condition under treatment with PLD2 inhibitor (200 nM) (b). PIN2-GFP seedlings were grown on 1/2 MS for 3 days and transferred to NP or LP conditions, or LP with PLD2 inhibitor (200 nM) for 7 days. Total proteins from root tips of treated seedlings were extracted and analyzed by using the antibody again GFP. The Actin was used as a loading control.

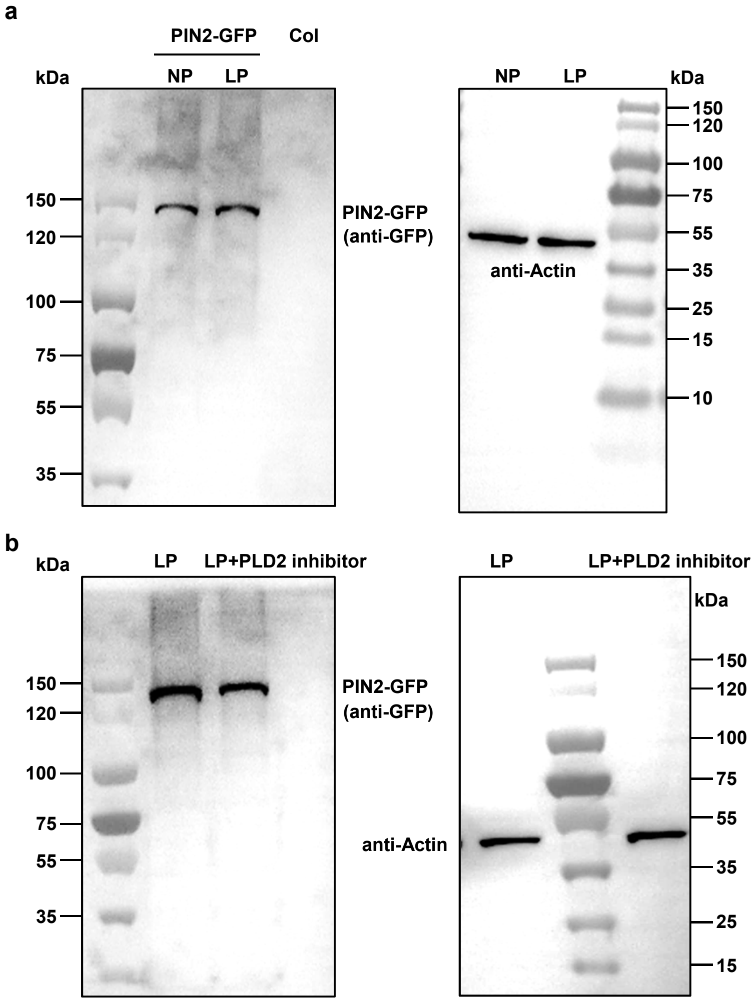

Fig. S4. Increased auxin level at root tips and elongation zones under LP condition. DR5:GFP or DR5:GUS seedlings were grown on 1/2 MS for 4 days and transferred to NP (1.25 mM Pi) or LP (0 mM Pi) condition, or LP with PLD2 inhibitor (200 nM) for 5 days. Fluorescence in root tips (a) or GUS activities in roots (b) were observed. The experiments were performed with three biological repeats and representative images were shown. Bar =100 μm.

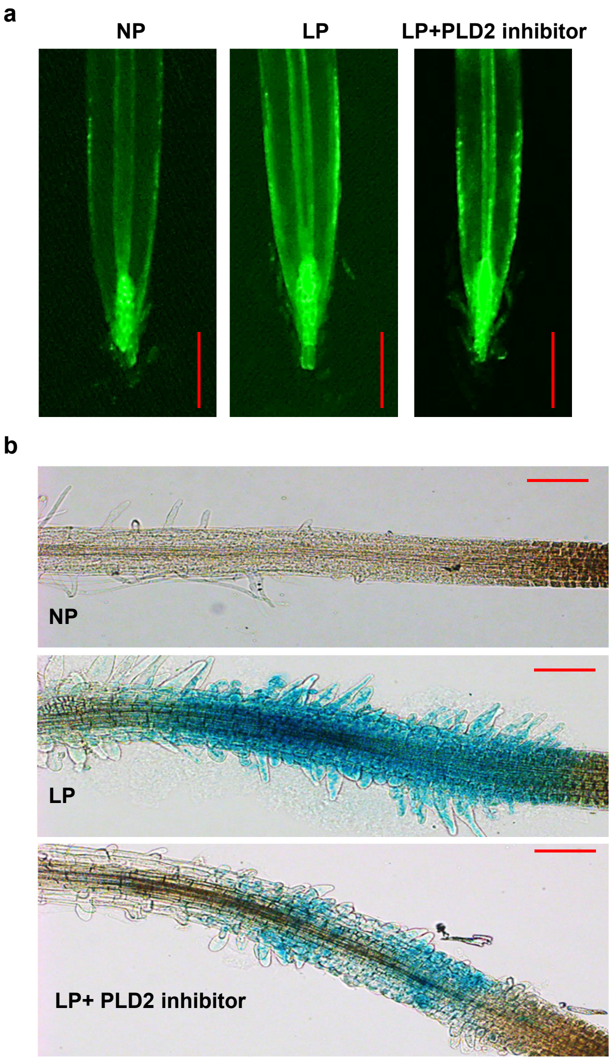

Fig. S5. Primary and lateral roots and root hairs are not altered in *pldζ2* and PLDζ2ox under LP condition. Col-0 and *pldζ2* seedlings were grown under NP (1.25 mM Pi) or LP (0 mM Pi) conditions for 3, 5, or 7 days and observed (a, bar = 1 cm). Primary root length, number of lateral roots and root hairs (per mm), and root hair length of Col-0, *pldζ2*, and PLDζ2ox seedlings were measured and calculated (b). The experiments were performed with three biological repeats, and data are presented as means ± SE ($n > 50$). Statistical analysis by two-tailed Student's *t*-test revealed no difference compared to Col-0.

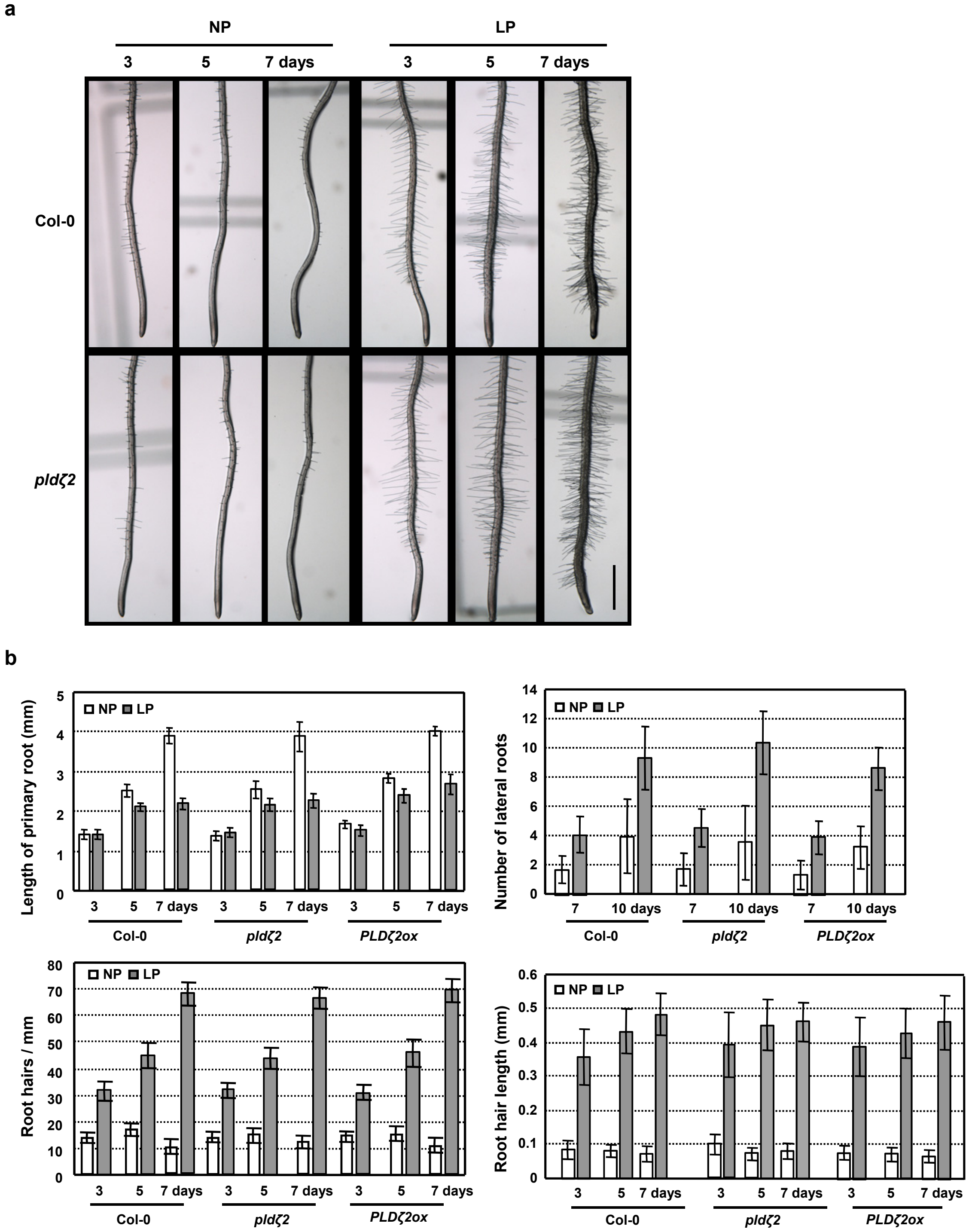

Fig. S6. Treatment with PLD2 inhibitor does not alter the primary root length or root hair number. Col-0 seedlings were grown under NP (1.25 mM Pi) or LP (0 mM Pi) conditions in the presence of a PLD2 inhibitor (0.2 μM, DMSO was used as a control) for 3, 5, and 7 days, and primary root length (upper) and root hair number (bottom) were measured. The experiments were performed with three biological repeats, and data are presented as means ± SE (*n* > 30). Statistical analysis by two-tailed Student's *t*-test revealed no differences among samples.

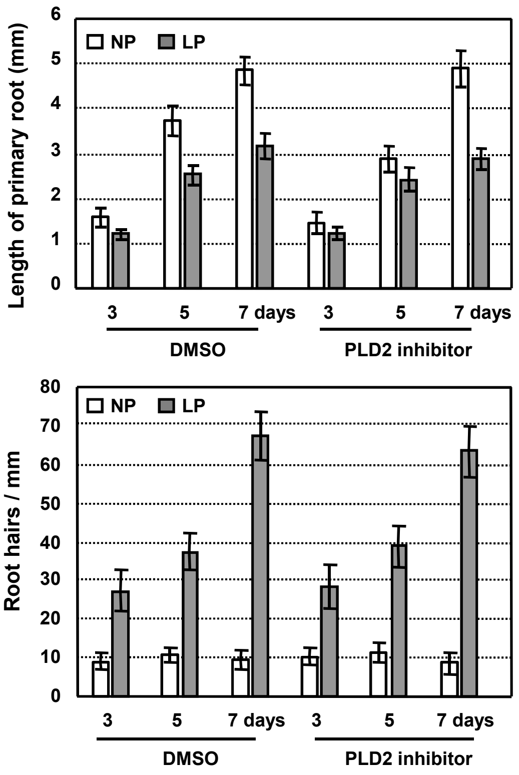

Fig. S7. Primary and lateral roots and root hairs are not altered in *snx1* under LP condition. Col-0 and *snx1* seedlings were grown under NP (1.25 mM Pi) or LP (0 mM Pi) conditions for 3, 5, or 7 days and observed (a, bar = 1 cm). Primary root length, number of lateral roots and root hairs (per mm), and root hair length of Col-0, *snx1*, and SNX1-ox seedlings (b). The experiments were performed with three biological repeats, and data are presented as means ± SE ($n > 50$). Statistical analysis by two-tailed Student's t-test revealed no difference compared to Col-0.

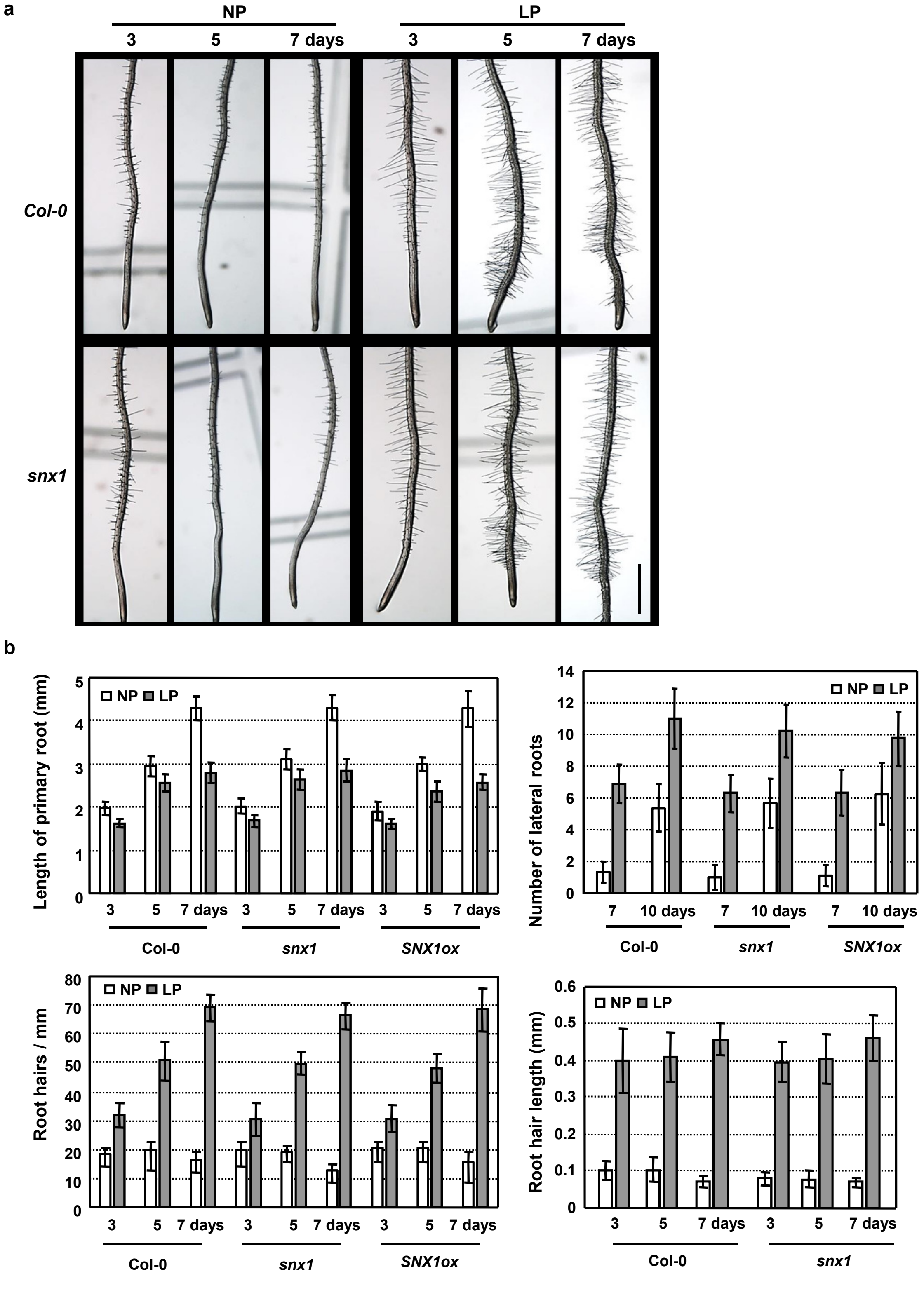

Fig. S8. Altered auxin levels at root elongation zones under LP condition with Wortmannin treatment. DR5:GFP or DR5:GUS seedlings were grown on 1/2 MS for 4 days and transferred to NP (1.25 mM Pi) or LP (0 mM Pi) conditions, or LP with Wortmannin (16.5 µM) for 5 days. Fluorescence in root tips (a) or GUS activities in roots (b) were observed. The experiments were performed with three biological repeats and representative images were shown. Bar =100 µm.

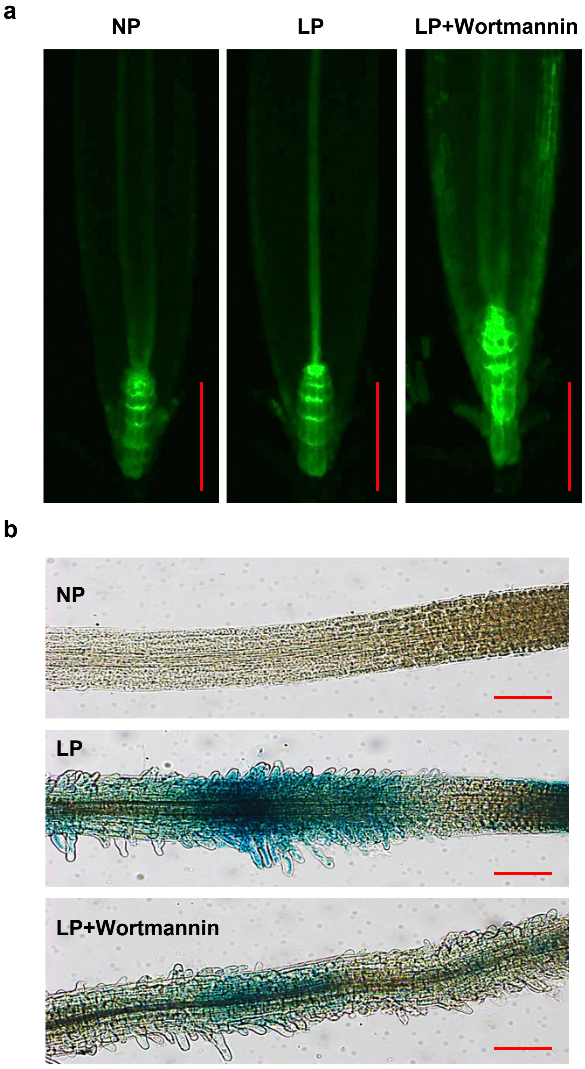

Fig. S9. LP condition suppresses the endocytosis but not the exocytosis of PIN2 and does not affect the endocytosis or exocytosis of SNX1 or SNX2a. Five-day-old WT seedlings expressing PIN2-GFP, pSNX1:SNX1-RFP, or pSNX2a:SNX2a-eGFP were treated with BFA (45 μM) for 0, 30, 60, or 90 min, followed by washout with 1/2 MS for 30, 60, or 90 min under both NP (1.25 mM Pi) or LP (0 mM Pi) conditions. The formation of BFA bodies was observed in SNX1-RFP (a), SNX2a-eGFP (b), and PIN2-GFP (c). Representative images are shown (bar = 50 μm).

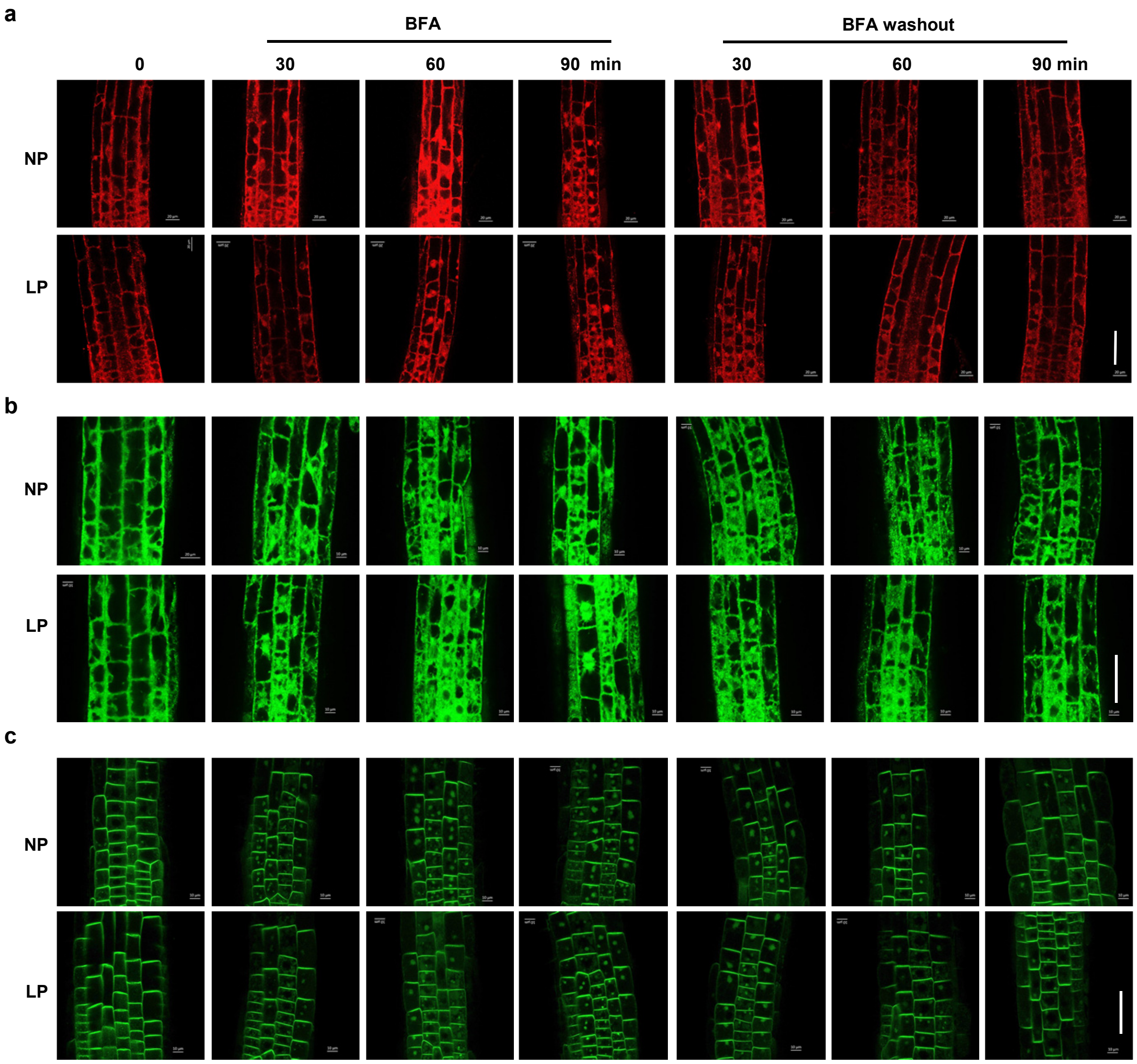

Fig. S10. Unaltered endocytosis of PIN2 under LP condition or *PLDζ2* and SNX1 deficiency. Five-day-old WT seedlings expressing PIN2-GFP in *snx1* mutant, in *pldζ2* mutant, or treated with PLD2 inhibitor (300 nM) were treated with BFA (45 µM) for 0, 30, 60, or 90 min, followed by washout with 1/2 MS for 30, 60, or 90 min, under both NP (1.25 mM Pi) or LP (0 mM Pi) conditions. The formation of BFA bodies was observed and representative images are shown (bar = 50 µm).

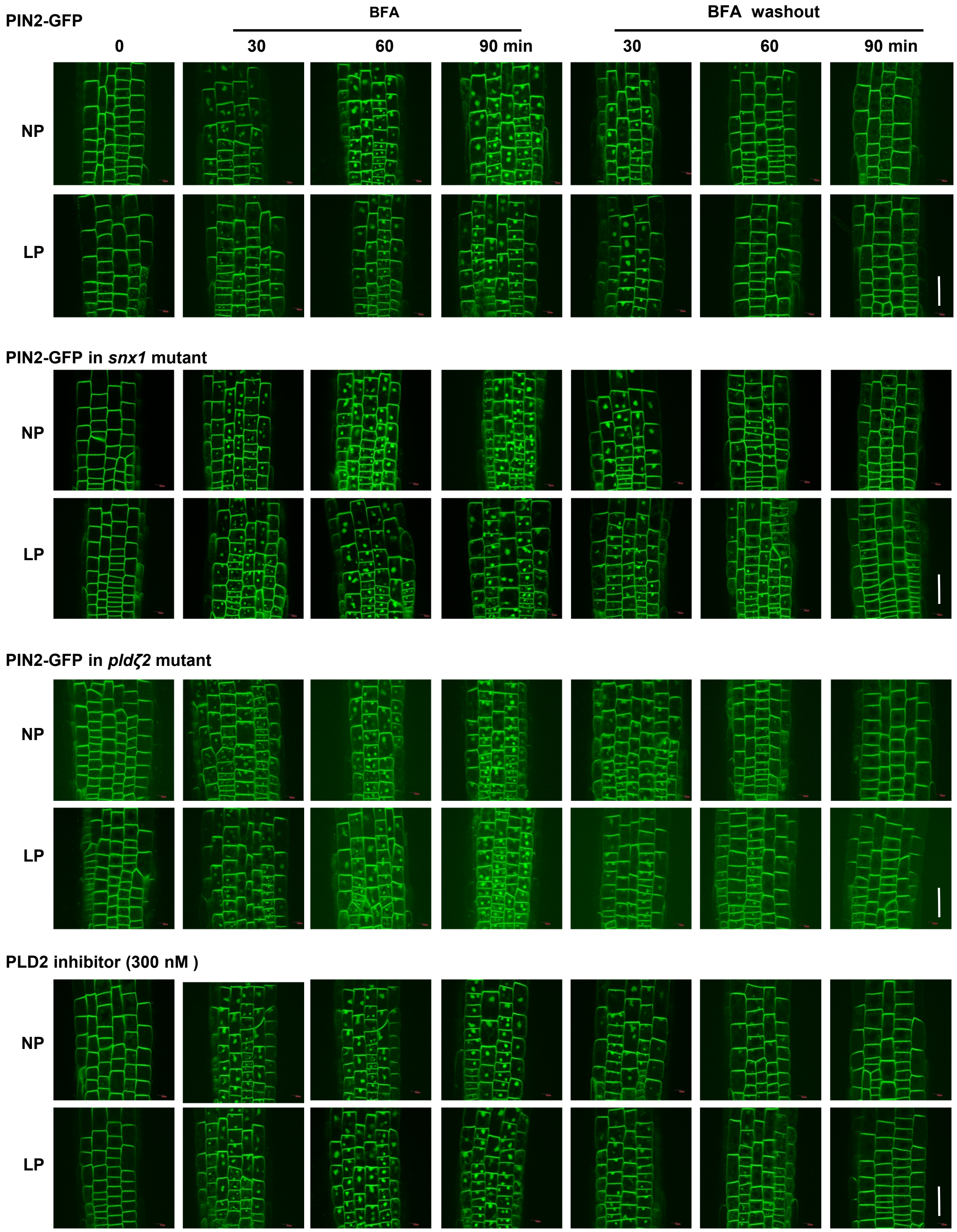

Supplement: Supplementary file 1 — Fig. S1 Primary and lateral root growth is not altered in pin2 under LP condition. Fig. S2 Visualization of accumulation of PIN2‐GFP protein to the vacuole. Fig. S3 PIN2 protein is accumulated under LP condition. Fig. S4 Increased auxin level at root tips and elongation zones under LP condition. Fig. S5 Primary and lateral roots and root hairs are not altered in pldζ2 and PLDζ2ox under LP condition. Fig. S6 Treatment with PLD2 inhibitor does not alter the primary root length or root hair number. Fig. S7 Primary and lateral roots and root hairs are not altered in snx1 under LP condition. Fig. S8 Altered auxin levels at root elongation zones under LP condition with Wortmannin treatment. Fig. S9 LP condition suppresses the endocytosis but not the exocytosis of PIN2 and does not affect the endocytosis or exocytosis of SNX1 or SNX2a. Fig. S10 Unaltered endocytosis of PIN2 under LP condition or PLDζ2 and SNX1 deficiency. [file NPH-226-142-s001.pdf]
